# Stress-induced metabolic exchanges between complementary bacterial types underly a dynamic mechanism of inter-species stress resistance

**DOI:** 10.1038/s41467-023-38913-8

**Published:** 2023-05-31

**Authors:** Kapil Amarnath, Avaneesh V. Narla, Sammy Pontrelli, Jiajia Dong, Jack Reddan, Brian R. Taylor, Tolga Caglar, Julia Schwartzman, Uwe Sauer, Otto X. Cordero, Terence Hwa

**Affiliations:** 1 Department of Physics, U.C. San Diego, La Jolla, CA 92093-0319 USA; 2 Institute of Molecular and Systems Biology, ETH Zürich, Zürich, Switzerland; 3 Department of Physics and Astronomy, Bucknell University, Lewisburg, PA 17837 USA; 4 Division of Biological Sciences, U.C. San Diego, La Jolla, CA 92093 USA; 5 Department of Civil and Environmental Engineering, MIT, Cambridge, MA 02139 USA

**Keywords:** Microbial ecology, Dynamical systems, Bacterial physiology, Bacterial systems biology

## Abstract

Metabolic cross-feeding plays vital roles in promoting ecological diversity. While some microbes depend on exchanges of essential nutrients for growth, the forces driving the extensive cross-feeding needed to support the coexistence of free-living microbes are poorly understood. Here we characterize bacterial physiology under self-acidification and establish that extensive excretion of key metabolites following growth arrest provides a collaborative, inter-species mechanism of stress resistance. This collaboration occurs not only between species isolated from the same community, but also between unrelated species with complementary (glycolytic vs. gluconeogenic) modes of metabolism. Cultures of such communities progress through distinct phases of growth-dilution cycles, comprising of exponential growth, acidification-triggered growth arrest, collaborative deacidification, and growth recovery, with each phase involving different combinations of physiological states of individual species. Our findings challenge the steady-state view of ecosystems commonly portrayed in ecological models, offering an alternative dynamical view based on growth advantages of complementary species in different phases.

## Abstract (web summary)

Microbes can cooperate and share resources via metabolic cross-feeding. Here, the authors show that excretion of key metabolites following acid stress provides a collaborative, inter-species mechanism of stress resistance.

## Introduction

Metabolic cross-feeding underlies many positive interactions between microbes^1–3^. Many well-studied examples of cross-feeding involve species that are *dependent* on each other for essential metabolic functions, including synthetic complementary auxotrophy^4–13^ and designated cross-feeding between symbionts^14,15^. The driving force for metabolic cooperation between such inter-dependent bacteria is clear since they lack the ability to generate essential metabolites themselves and must obtain them from other species to grow.

Many bacteria in nature, however, are prototrophic, or “free living”—that is, they can grow on simple substrates without the help of others^16,17^. Recent studies indicated that substantial cross-feeding of diverse metabolites supported the coexistence of many species of naturally occurring, free-living bacteria, even in synthetic bacterial communities provided with just one or a few substrates for growth^18–20^. In these cases, metabolic cross-feeding is recognized as essential for community diversity, since without them all species would compete directly for the few externally-provided nutrients and only a few species that grow well on those nutrients will survive according to the Competitive Exclusion Principle^21,22^. However, for metabolic cross-feeding to relieve the constraint of Competitive Exclusion, a *substantial* part of the externally-provided nutrients will need to be excreted in forms of other metabolites, and subsequently taken up by other species for their growth. For example, if 50% of the final community biomass is comprised of species that do not directly consume the environmentally-provided carbon source, this would require the species directly consuming the provided carbon source to excrete more than 50% of what it takes up. However, if carbon is the growth-limiting nutrient for this community, what physiological rationale is there for free-living bacteria to excrete such a large fraction of this limiting nutrient when they need it for their own growth?

Free-living bacteria can be forced into excreting large amounts of metabolites, via e.g., internal bottlenecks created by genetic manipulations^23^, the design and attainment of which is an important goal of synthetic biology^24–26^. Naturally-occurring free-living bacteria are generally not known to excrete large amounts of endogenous metabolites during their own growth; however, except for a few well-documented cases, including overflow metabolism during aerobic fermentation^27–29^, the excretion of nitrate/nitrite during anaerobic denitrification^30,31^, and complex cascades of fermentation product removal in anaerobic digesters^32,33^. Even in those cases, the amounts of excretion measured are not overwhelming. For example, for *E. coli* growing aerobically on glucose, ~5 mM/OD of glucose is taken up while ~2–3 mM/OD of acetate is excreted, placing the fraction of excreted carbon below 20%. The prevalence of metabolic cross-feeding between free-living bacteria^18–20^ thus suggests the existence of additional driving forces that we currently know little about.

In this study, we reveal an important physiological driving force for substantial metabolic excretion by naturally-occurring, free-living bacteria. We find that stressed, growth-arrested bacteria convert externally-provided carbon substrates into valuable central carbon metabolites and subsequently excrete them almost completely, and that these excreted metabolites are required for other growth-arrested species in the community to resume growth and relieve stress, ultimately restoring the growth of the whole community. This collaborative inter-species stress relief mechanism can occur between species taken from vastly different environments, indicating that it is not a result of selection in specific environments. Instead, this interaction is attributed to a fundamental complementarity between free-living bacteria with opposing modes of metabolism, with both modes needed to overcome stress.

The stress under study here arises from the accumulation of weak organic acids, e.g., acetate, which are commonly encountered in many environments, from the gut to bioreactors^34–42^. Weak organic acids are excreted during anaerobic growth^27^, but also aerobically under iron limitation^43^, as well as in favorable growth conditions^28,29^. The excreted acids become toxic to both the acid-excreting and acid-consuming bacteria when the environmental pH drops to the level of the acids’ dissociation constants, ~5 for organic acids such as acetate^44,45^ We reveal that during such acid stress, an additional layer of metabolic exchange occurs transiently between the growth-arrested acid excreters and acid eaters—“acid-induced cross-feeding”—which is necessary for the acid eaters to consume the organic acids at low pH and thereby detoxify the environment. Based on quantitative, systematic investigations, we will first describe acid-induced cross-feeding in a case of rapid acetate accumulation during the aerobic growth of a co-culture of marine bacteria. We will then show that the same process of stress relief occurs in co-cultures comprising soil and enteric bacteria, and for different mechanisms of acetate accumulation.

## Results

### Simple acetate cross-feeding in strong buffer

**Fig. 1:**
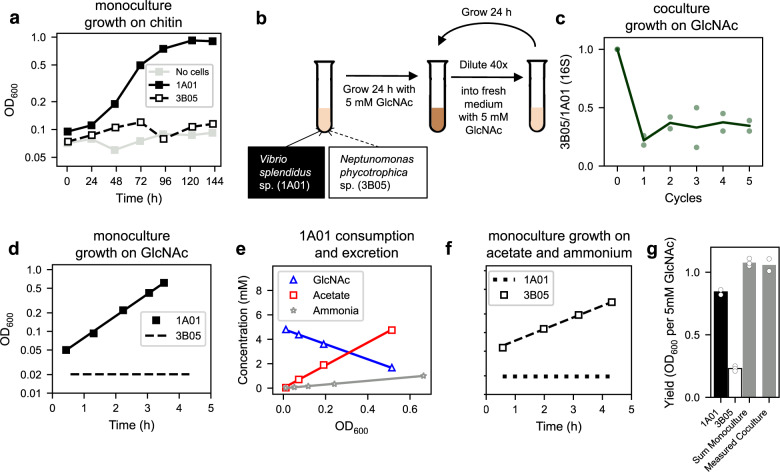
Growth of 1A01 and 3B05 in strongly-buffered medium. *Vibrio splendidus* sp. 1A01 and *Neptunomonas phycotrophica* sp. 3B05 were cultured individually or together in media with various defined carbon and nitrogen sources, with 40 mM HEPES buffer and 0.35 M NaCl; see details of growth medium and conditions in “Methods”. **a** 1A01 and 3B05 were grown in monoculture with 0.2% w/v chitin chips as the sole carbon source. The effect of residual small chitin pieces in the culture is shown by a ‘no cell’ control (gray squares). **b** Growth-dilution cycles of 1A01 and 3B05 co-culture with 5 mM GlcNAc as the sole carbon and nitrogen source, with a 24-h cycle and 40x dilution after each cycle. **c** Ratio of 3B05 to 1A01 based on 16S reads at the end of each cycle (Supp. Fig. 1), starting from 1:1 mixture of exponentially-growing cells each at OD_600_ = 0.01. Data from each replicate is shown as open green circles. **d** Steady-state growth of 1A01 (filled black squares) with 5 mM GlcNAc as the sole carbon and nitrogen source. The solid line indicates an exponential fit to the 1A01 growth curve. 3B05 was not observed to grow on GlcNAc; the dashed black line indicates the average OD. **e** Depletion of GlcNAc (blue triangles) and accumulation of acetate (red squares) and ammonium (gray stars) during steady-state growth of 1A01 on GlcNAc. The solid lines indicate linear fits, giving the inverse biomass yield on GlcNAc, and acetate and ammonium excretion yields. **f** Steady-state growth of 3B05 (open black squares) on 60 mM sodium acetate as the sole carbon source and 10 mM NH_4_Cl as the sole nitrogen source. The dashed black line indicates an exponential fit to the 3B05 growth curve. 1A01 was not observed to grow on acetate and ammonium; the dotted black line indicates the average OD. The values of the best-fit parameters in (**d**), (**e**), and (**f**), along with the standard deviations of the fits, are summarized in Supplementary Table 1. **g** Filled black bar indicates the monoculture yield of 1A01 (in OD_600_) on 5 mM GlcNAc. Open bar indicates the monoculture yield of 3B05 on 7.4 mM acetate (the amount of acetate excreted by 1A01 after growth on 5 mM GlcNAc, **e**). Gray left bar indicates the sum of the yields of 1A01 and 3B05 shown to the left, and right gray bar indicates the measured yield of the 1A01-3B05 co-culture on 5 mM GlcNAc. Measurement variability is ±0.002 OD_600_ based on repeated measurements of the same culture sample. Data from replicates are denoted by open circles in each case. All data in this and other figures are provided in the Source Data file.

This study started with the characterization of *Vibrio splendidus* sp. 1A01 and *Neptunomonas phycotrophica* sp. 3B05, two species co-isolated from a chitin enrichment culture of coastal ocean water^46^, to study simple cross-feeding between two natural, free-living strains of bacteria. When cultured alone on chitin, 1A01 grew, but 3B05 did not (Fig. 1a). To investigate possible reasons for the presence of 3B05 and many other non-chitin-degrading bacteria in the enrichment culture^46^, we grew these two strains together using N-acetyl-glucosamine (GlcNAc), the monomer of chitin, as the sole carbon and nitrogen source, in defined, minimal medium strongly buffered at pH = 8, the canonical pH of sea water^47^; see “Methods”. After inoculating 1A01 and 3B05 at equal ratio, the co-culture was left to grow for 24 h, then diluted 40-fold into fresh medium. Such 24-h growth-dilution cycles were repeated for several days (Fig. 1b). Before each dilution, the abundance of each species was monitored using 16S PCR^48^ (Supp. Fig. 1). The two species were found to coexist stably, settling after a few cycles to a ratio 3B05:1A01 ≈ 1:3 by 16S abundance (Fig. 1c).

To determine the mechanistic basis of the coexistence of these two species on GlcNAc, we quantified the growth and uptake/excretion characteristics of each species in monoculture. Only 1A01 grew in monoculture with GlcNAc as the sole carbon and nitrogen source (Fig. 1d). Analysis of the culture medium using HPLC (readily detecting >10 ${{{{{\rm{\mu }}}}}}{{{{{\rm{M}}}}}}$ of common carbohydrates and amino acids; see “Methods”) found substantial accumulation of acetate and ammonium (Fig. 1e). A closer examination of the excretion data (Supp. Fig. 2) suggests that the acetate liberated in the conversion of GlcNAc to glucosamine^49^ was directly released into the medium, in addition to the acetate released due to overflow metabolism during rapid growth on glucose^29^.

The substantial excretion of acetate and ammonium by 1A01 in monoculture suggested that 3B05 might be growing on these carbon and nitrogen sources in the co-culture. As a first test, we grew 1A01 and 3B05 as monocultures on acetate and ammonium and found that only 3B05 grew (Fig. 1f). The results suggest that simple commensalism between 1A01 and 3B05 underlies the coexistence found in Fig. 1c. Indeed, the yield attained by the co-culture can be quantitatively explained by the sum of the two monoculture yields measured during exponential, steady-state growth (Fig. 1g).

### Acetate cross-feeding is insufficient for coexistence in a weak buffer

**Fig. 2:**
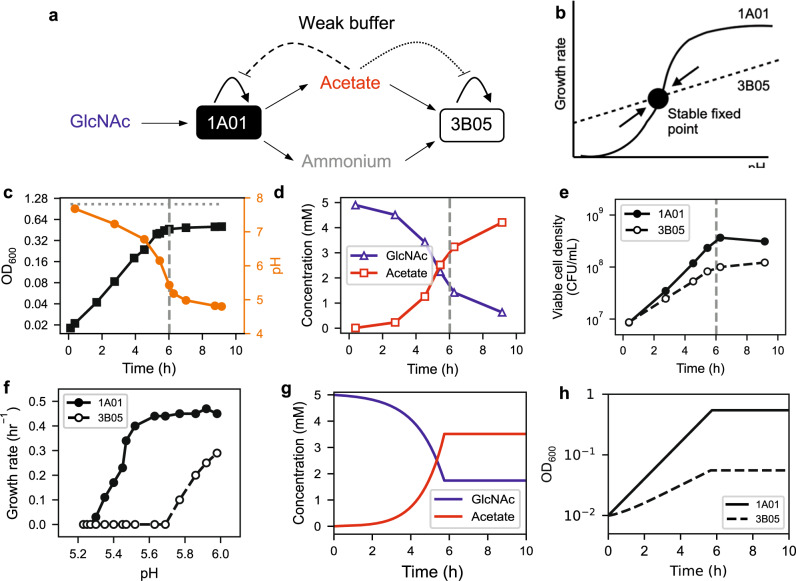
1A01 and 3B05 co-culture in weak bicarbonate buffer. **a** Solid arrows indicate schematic of acetate cross-feeding based on single–strain characteristics derived from Fig. 1. In a weak buffer, acetate excretion will reduce the pH and inhibit cell growth as indicated by the dashed and dotted lines. **b** A canonical scenario of syntrophy is realized if the growth-inhibiting effect exerted on the acid excreter (1A01, solid line) is stronger than that on the acid eater (3B05, dashed line) as pH drops. The intersection of these two lines is the fixed point describing a stable, exponentially-growing co-culture. To test this scenario, we grew the 1A01 and 3B05 co-culture in 5 mM GlcNac in weak buffer (2 mM bicarbonate), inoculated at 1:1 ratio. **c** shows the OD (black squares) and pH (orange circles), with the horizontal dotted line indicating the final OD reached by the same co-culture grown in strong buffer (Fig. 1g). **d** shows the GlcNAc (blue triangles) and acetate (red squares) concentrations in the medium. **e** shows the viable cell density (Supp. Fig. 3) for 1A01 (filled circles) and 3B05 (open circles). The vertical dashed line in (**c**)–(**e**) indicates the time when the increase in OD ceased according to (**c**). **f** Dependences of the growth rate of 1A01 (solid circles) and 3B05 (open circles) on the medium pH. Cells were grown in minimal medium buffered by 10 mM MES with different ratios of the acid and base form to obtain the desired pH. Glycerol was used as the sole carbon source as both strains grew on it and neither strain excreted acetate or other fermentation products which would have changed the medium pH during the course of experiment. The data shows that 3B05 is more sensitive to pH than 1A01, hence precluding the scenario of a stable, exponentially-growing co-culture depicted in (**b**). We developed a simple quantitative model (Supplementary Note 1) for the co-culture dynamics using single-strain characteristics summarized in Supplementary Table 1 and acid response data shown in (**f**). **g** Model output on the concentrations of GlcNAc (blue line) and acetate (red line) agree quantitatively with those measured in (**d**) up to the time of growth arrest. **h** Model output on the densities of 1A01 and 3B05 cells (solid and dashed lines) agree quantitatively with the observed viable cell densities shown in (**e**). The model also correctly predicted growth arrest to occur about 6 h after inoculation (position of the vertical dashed lines in (**c**)–(**e**), with about one-third of the initial GlcNAc still remaining at that time. This simple model does not predict what occurs after the growth arrest, which is the focus of the rest of the study.

One important feature of the co-culture described above is the high buffer capacity (40 mM HEPES) used, which fixed the medium pH despite acetate accumulation and enabled us to focus solely on nutrient consumption and cross-feeding. A scenario of broad ecological relevance is one in which the medium is acidified by the excreted organic acids, as many natural environments including the ocean are weakly-buffered^50–52^, and acidification (i.e., pH drop) can easily occur when the excreted acid reaches the order of the buffer capacity of the environment. Thus, in the ocean which is buffered by ~2 mM bicarbonate (primarily from equilibration with atmospheric CO_2_^52^), acidification would occur when the excreted acetate reaches ~2 mM. As bacterial growth is generally inhibited at reduced pH, especially in the presence of weak organic acids such as acetate^44,45,53^, and the presence of acid eaters such as 3B05 would alleviate acidification, the relationship between 1A01 and 3B05 changes from a commensal one for the co-culture in a strong buffer to a syntrophic one in a weak buffer; see illustration in Fig. 2a. Assuming that 3B05 is less affected by reduced pH than 1A01 in a weakly-buffered co-culture, the growth of 3B05 on acetate would limit the acetate buildup and hence the drop in medium pH, resulting in a canonical syntrophy scenario in which the two species grow exponentially at the pH where the growth rate of the two species matches; see Fig. 2b.

However, when we grew 1A01 and 3B05 together in 2 mM bicarbonate^52^, the co-culture stopped growing after ~6 h (black squares, Fig. 2c) where the pH plummeted (orange circles), reaching a final OD which is less than half of that reached in the strong buffer (horizontal dotted line). This is consistent with our analysis of the medium, which found GlcNAc dropping and acetate accumulating in the medium (blue triangles and red squares, respectively, in Fig. 2d), such that the total carbon content of GlcNAc and acetate is about one-half of the starting amounts at the time of the growth arrest (vertical dashed line). Also, the acetate concentration in the medium exceeded the buffer capacity (2 mM) at ~5 h, at which point pH started dropping rapidly, followed soon by growth arrest. Interestingly, GlcNAc concentration continued to decrease and acetate continued to increase after OD stopped increasing after 6 h, suggesting residual metabolic activity in the non-growing co-culture which we will delve into below.

To see why 3B05 was unable to prevent acetate build-up as depicted in the classic syntrophy scenario (Fig. 2a, b), we characterized the densities of viable 1A01 and 3B05 cells using plating (Supp. Fig. 3). Our data shows that both species stopped growing (Fig. 2e) at around the vertical dashed line where the pH dropped to below 5.5 (Fig. 2c). We tested for the steady-state growth of these species individually at various fixed pH and found 3B05 to be more sensitive to reduced pH than 1A01 (Fig. 2f), contrary to the scenario of Fig. 2b canonically assumed^54^. Thus, given that 1A01 grows faster on GlcNAc than 3B05 grows on acetate (Fig. 1d, f), acetate accumulation in the medium and the resulting pH drop and growth arrest of both species is inevitable. The dynamics of the co-culture observed here are quantitatively captured by a simple metabolic model^55^ (Fig. 2g, h)^55^, using single-strain characteristics obtained from the two monocultures (Supp. Table 1) without ad hoc parameter fitting; see Supplementary Note 1.

**Fig. 3:**
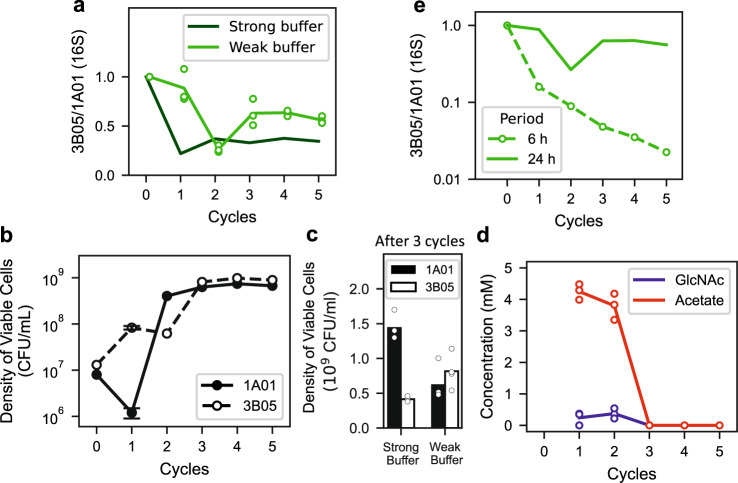
Coexistence of 1A01 and 3B05 in growth-dilution cycles in the weak buffer. 1A01 and 3B05 were co-cultured in growth-dilution cycle (Fig. 1b), with 5 mM GlcNAc as the sole carbon and nitrogen source in the weak buffer (2 mM sodium bicarbonate). **a** Ratio of 3B05 to 1A01 based on 16S reads at the end of each cycle, starting from 1:1 mixture of exponentially growing cells each at OD_600_ = 0.01. The results for each biological replicate is shown as the open green circles. The light green line connects the average over the replicates after each cycle. The average obtained for the strong buffer case (Fig. 1c) is reproduced here (the dark green line) for comparison. **b** Viable 1A01 and 3B05 cells in a co-culture can be distinguished by plating (Supplementary Fig. 3). Densities of viable 1A01 cells (filled circles) and 3B05 cells (open circles) obtained at the end of each 24-h cycle are shown for a co-culture passaged through five consecutive cycles in the weak buffer. The standard error of the mean for the first cycle is based on 3 biological replicates. **c** Composition of the co-culture after three or more 24-h growth-dilution cycles in the strong HEPES buffer (left bars) and in the weak bicarbonate buffer (right bars); data from individual replicates are shown as open circles. **d** Concentrations of GlcNAc (blue symbols) and acetate (red symbols) in the medium, measured at the end of each 24-h cycle for the co-culture grown in weak buffer. Results from each biological replicate are shown as open circles; the lines connect the average of the data at each cycle. **e** Ratio of 3B05 to 1A01 based on 16S reads, collected at the end of each cycle of 6-h growth-dilution experiments in the weak buffer (open green circles, connected by the dashed green line). For ease of comparison, the average result obtained for the co-culture in 24-h cycle in the weak buffer (**a**) is reproduced as the solid green line.

Because 3B05 grew less than 1A01 during the 6-h period prior to the growth arrest (Fig. 2e), we expected it to be depleted from the co-culture if the growth-dilution experiment of Fig. 1b was repeated in the weak buffer. Contrary to our expectation, however, coexistence remained, as measured by 16S ratio (light green symbols, Fig. 3a) and by cell count (Fig. 3b) at the end of each cycle. The co-culture settled after a few cycles to a stable composition favoring 3B05 (as opposed to the strong buffer case (Fig. 3c) where 1A01 is favored). Moreover, measurements of GlcNAc and acetate concentrations in the medium at the end of each cycle showed complete consumption of GlcNAc with no acetate accumulation once the co-culture stabilized after a few cycles (Fig. 3d). To look for possible syntrophic interaction that might have escaped our analysis, we repeated the growth-dilution experiment with 6-h cycles to maintain the co-culture in exponential growth, mimicking a rapidly diluting chemostat (since each species grew exponentially in the co-culture during the first 6 h, see Fig. 2e). 3B05 is seen to deplete rapidly as expected (Fig. 3e, open circles). Thus, the coexistence observed in the 24-h growth-dilution experiment resulted from some syntrophic interaction that occurred outside of the exponential growth phase.

The big drop in 1A01 viability after the first 24-h cycle in the weak buffer (Fig. 3b) resulted from rapid cell death after GlcNAc was depleted (Supp. Fig. 4). Because the death of a rapidly proliferating species can promote coexistence^56^, we examined the possibility that the coexistence observed in Fig. 3 arose from the preferential death of 1A01. However, adding cell death to our metabolic model could not account for the coexistence observed (Supplementary Note 2), because death-mediated coexistence would give a species ratio biased strongly towards 3B05 (around 100×) and leave a substantial amount of nutrient unconsumed, while our data show comparable counts of 1A01 and 3B05 (Fig. 3a–c) and the complete depletion of nutrient (Fig. 3d) once the co-culture stabilized after a few cycles. We also examined the question of whether the coexistence might have resulted from mutation and selection during the growth-dilution cycles. However, repeating the growth-dilution experiment using clones isolated from the end of 5 consecutive growth-dilution cycles yielded very similar results (Supp. Fig. 5), indicating that evolution is not a concern over the course of the growth-dilution experiments.

### Growth and metabolite dynamics in the stable cycle

**Fig. 4:**
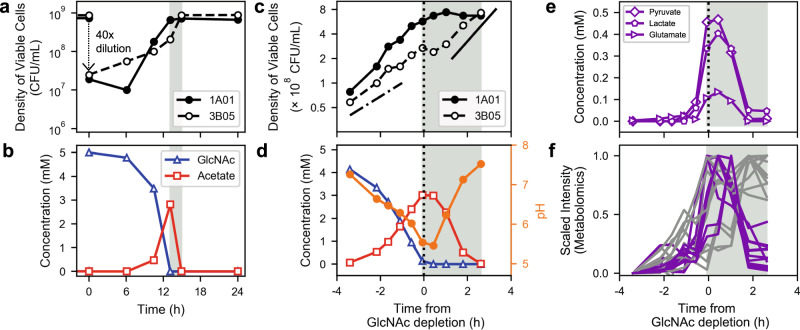
Cross-feeding in the stable cycle of the coculture in weak buffer. Measurements of various quantities of the coculture throughout the fifth 24-h growth-dilution cycle. **a** The viable counts of 1A01 and 3B05 cells. The value shown was the average of three measurements on the same sample from a single co-culture; the error between these measurements was less than the size of the data marker. **b** The concentrations of GlcNAc and acetate in the medium from the same co-culture measured in (**a**). The measurement variability for the determination of sugar, organic acid, or amino acid concentrations by HPLC was ~2% on the basis of repeated measurements of the same spent media sample. The light gray regions in (**a**) and (**b**) indicate the period when 3B05 continued to grow after GlcNAc depletion. In (**c**)–(**f**), the duration around the “acetate peak” was densely sampled using a protocol that mimicked the stable cycle; see “Methods”. The data from all four panels were measured on the same coculture. The pH measurement was accurate to ±0.02 pH unit on the basis of repeated measurements of pH standards. The dotted vertical line at time “0” indicates the time of GlcNAc depletion, around 12 h into the cycle (**b**). Gray-shaded regions are the same as those in (**a**) and (**b**). The same symbols are used in (**c**) and (**d**) as in (**a**) and (**b**). In (**c**) the dashed-dotted line indicates exponential growth of 3B05 at rate ~0.35/h before the acetate peak; the solid line indicates a growth rate ~0.55/h after the acetate peak. The filled orange circles in (**d**) indicate the culture pH (right vertical axis). **e** Concentrations of pyruvate, lactate, and glutamate in the medium as measured by HPLC. **f** Scaled intensities of metabolites in the medium as measured by untargeted metabolomics; see “Methods”. Metabolites consumed (defined as those with the scaled intensity of the last timepoint <0.5) are plotted in purple. Other detected metabolites are plotted in gray. Identities of the metabolites are given in Supplementary Table 2 and their values are provided in the Source Data file.

To find mechanisms enabling both coexistence and full consumption of carbon, we analyzed the dynamics of the co-culture in the “stable cycle”, several cycles after the initial inoculation when the levels of the two species and the carbon concentrations stabilized (Fig. 3a–d). We measured the viability of 1A01 and 3B05 (Fig. 4a) and concentrations of GlcNAc and acetate (Fig. 4b) at various times during the stable cycle. The dynamics observed were strikingly different from that in the first 24-h: First, acetate in the medium was high only briefly in the middle of the stable cycle (red squares, Fig. 4b), with all of it consumed shortly after 1A01 stopped growing. Given the prolonged exposure to high acetate in the first 24-h and the rapid death of 1A01 in high acetate (Supp. Fig. 4), the maintenance of 1A01 viability in the stable cycle can be attributed to the rapid disappearance of acetate. Next, the growth of 3B05 (open circles, Fig. 4a) surged when acetate reached ~3 mM (shaded region), even though 3B05 stopped growing when acetate reached a similar level in the first cycle (Fig. 2d, e). Moreover, during the first 6-h of the stable cycle, despite the absence of acetate and the availability of GlcNAc in the fresh medium (Fig. 4b), 1A01 did not grow yet 3B05 managed to grow in that same condition (Fig. 4a). These puzzles are addressed below by analyzing monocultures in conditions mimicking various phases of the stable cycle. The results will reveal how acetate is removed and species coexistence is maintained in the stable cycle.

### Growth of 3B05 is aided by excretants of growth-arrested 1A01

To determine the cause of the surge in 3B05 towards the end of co-culture growth in the stable cycle, we measured cell viability and analyzed the spent medium at many time points during the period when acetate peaked (Fig. 4c). The dense sampling revealed that the growth of both species dropped as acetate accumulated, driving pH below 6 (red squares and orange circles, Fig. 4d). This growth inhibition is referred to here as “acetate stress” or more generally as “acid stress”. Unlike the first 24-h (Supp. Fig. 4a, b) where substantial GlcNAc remained at the onset of growth arrest, the growth arrest in the stable cycle coincided approximately with the complete exhaustion of GlcNAc (blue triangles, Fig. 4b, d).

Analysis of the medium by HPLC revealed that, in addition to acetate, several other metabolites, namely pyruvate, lactate, and glutamate, accumulated to high concentrations starting from 30 to 60 min before GlcNAc was exhausted (Fig. 4e). The ensuing disappearance of these metabolites (gray-shaded region) coincided with the depletion of acetate and the recovery of the pH (Fig. 4d), and the surge of 3B05 growth (open circles, Fig. 4c), while the density of 1A01 remained constant during this period (filled circles, Fig. 4c). During its surge, 3B05 grew at a rate substantially larger than on acetate alone (compare solid and dash-dotted lines in Fig. 4c). This faster growth rate is consistent with the growth rate of 3B05 on a mixture of acetate, lactate, pyruvate, and glutamate at normal pH (Supp. Fig. 6), suggesting that the surge of 3B05 was aided by these additional metabolites in the medium. The consumption of these additional metabolites would also account for the higher composition of 3B05 reached in the stable cycle in weak buffer compared to that in the strong buffer (Fig. 3c).

**Fig. 5:**
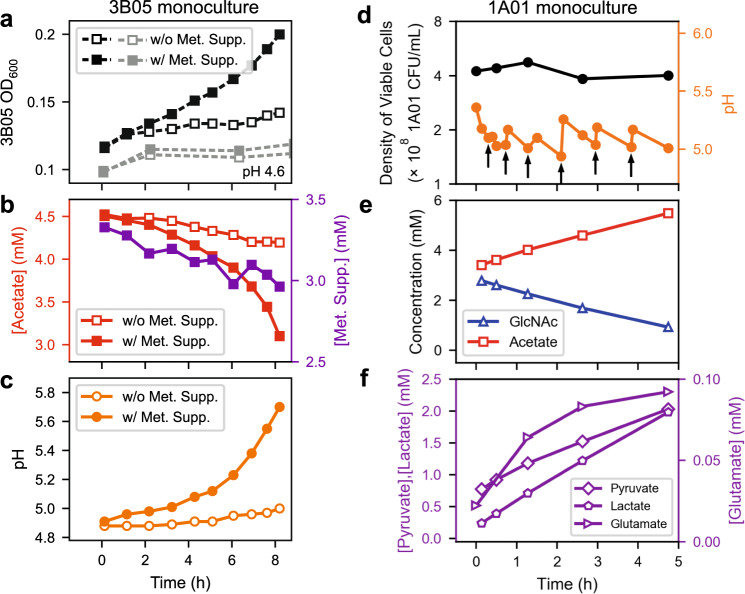
Key physiological and metabolic characteristics of 1A01 and 3B05 monocultures under acetate stress. 3B05 was precultured alone in strongly buffered acetate medium. Then the culture was washed and transferred to a weakly buffered medium (2 mM NaHCO_3_) with 4.5 mM acetic acid (with pH = 4.9), supplemented with (filled symbols) or without (open symbols) the addition of pyruvate, lactate, and glutamate (referred to collectively as a Supplement, or “Supp”). **a** OD, with the gray symbols showing results from the same experiment starting at a lower pH (4.6, by the addition of HCl). The measurement variability for the determination of OD_600_ was ±0.002 on the basis of repeated measurements of the same culture sample. **b** Acetate (left axis) and the sum of pyruvate, lactate, and glutamate concentrations ([Supp], right axis) in the medium of the two cultures described in (**a**), and (**c**) pH of the medium. The measurement variability for the determination of acetate, pyruvate, lactate, and glutamate concentrations by HPLC was ~2% on the basis of repeated measurements of the same spent media sample. The pH measurement was accurate to ±0.02 pH unit on the basis of repeated measurements of pH standards. Exponentially growing 1A01 monoculture was initiated at an OD_600_ of 0.2 and grew in steady-state in GlcNAc medium with the weak buffer (2 mM NaHCO_3_) until acetate excretion dropped the pH to ~5 where OD_600_ reached 0.45, corresponding to viable cell density of ~4 × 10^8^ CFU/mL. The pH was then maintained in a narrow pH range by manually titrating with 0.1 M NaHCO_3_. **d** Viable cell count (black circles) and pH (orange circles); the arrows indicate times at which NaHCO_3_ was added. **e** Concentrations of GlcNAc (blue triangles) and acetate (red squares) in the medium. **f** Concentrations of pyruvate, lactate, and glutamate in the medium.

This surge of 3B05 is a crucial phase of the co-culture dynamics despite its short duration (of ~2 h), because the density of 3B05 nearly quadrupled. To understand how 3B05 managed to grow during the surge period when the pH was initially low, while it did not grow for pH <5.7 in the monoculture (open circles, Fig. 2f), we grew the 3B05 monoculture in medium acidified by acetate, with and without the supplement of lactate, pyruvate, and glutamate, the metabolites which accumulated significantly during the surge (Fig. 4e). 3B05 only grew with the supplement (filled black squares, Fig. 5a), accompanied by the uptake of both acetate and the supplements (filled squares, Fig. 5b) and by pH recovery (filled circles, Fig. 5c). Thus, these supplements relieved the growth inhibition experienced by 3B05 when it was with acetate alone (open symbols, Fig. 5a–c).

To clarify where these metabolites came from, we maintained an acetate-inhibited 1A01 monoculture in GlcNAc at pH between 5 and 5.5 (orange circles, Fig. 5d), to capture the conditions during the acetate peak in the stable cycle of the co-culture where pH dropped below 5.5 (Fig. 4d). In this high acetate, low pH condition, 1A01 did not grow (black circles, Fig. 5d) but GlcNAc was gradually depleted while acetate, lactate, pyruvate, and glutamate accumulated in the medium (Fig. 5e, f), in contrast to the accumulation of just acetate under normal pH (Fig. 1e). The additional metabolites were not mainly from dead/lysed cells because 1A01 viability did not drop while these metabolites were accumulating (Fig. 5d); more importantly, the amount of carbon released (1.2 mM pyruvate, 1.8 mM lactate, 2 mM acetate, totaling ~13 mM of C-atoms in 5 h) was comparable to that contained in the amount of GlcNAc consumed ($\lesssim$2 mM) during this period (Fig. 5e, f). Thus, these metabolites were actively converted from GlcNAc by the growth-arrested 1A01 cells under acetate stress. (The amount of glutamate was negligible compared to pyruvate and lactate and not included here and below.) Untargeted metabolomic analysis^57,58^ of the spent medium of 1A01 monoculture during self-acidification showed the increase of numerous other metabolite features in addition to those already mentioned (Supp. Fig. 7). To see whether the corresponding metabolites may also be cross-fed in the co-culture, we analyzed the spent media collected during the acetate peak using untargeted metabolomics. Many metabolite features were found (purple curves, Fig. 4f) with similar dynamics as those exhibited by lactate, pyruvate, and glutamate in Fig. 4e. Altogether, these data suggest that, in addition to acetate, diverse metabolites were excreted by 1A01 and cross-fed to 3B05, although at a quantitative level, pyruvate, lactate, and acetate were the most dominant ones.

### Approach to the stable cycle

**Fig. 6:**
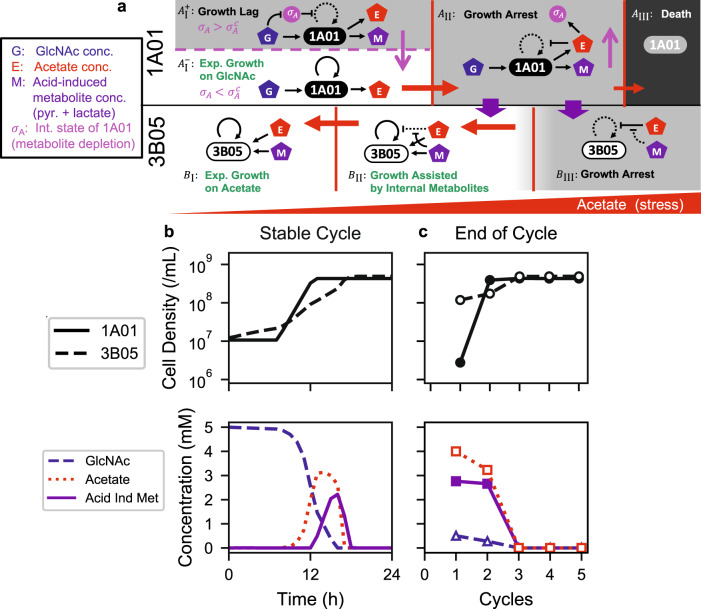
Model of acid-induced cross-feeding between 1A01 and 3B05 and the resulting population dynamics in 24-h growth-dilution cycles. **a** We describe the cross-feeding dynamics by a consumer-resource model outside of steady-state growth. The model involves the densities of 1A01 and 3B05 cells and the concentrations of GlcNAc (G), acetate (E), and acetate-induced metabolites (M, to be interpreted as the sum of pyruvate and lactate concentrations in the medium). We also introduce an additional variable ${\sigma }_{A}$ to describe the internal state of 1A01 due to the depletion of other metabolites such as aspartate and glutamate (Supp. Figs. 8, 10). The key feature of our model is that the growth/death rate of the two species and the rates of uptake/excretion of the metabolites G, E, and M by the two species are dependent on the degree of acetate stress (E) and 1A01’s internal state (${\sigma }_{A}$). As illustrated in the schematic, we approximate these dependences by switching between several distinct forms of the rate functions depending on the values of ${\sigma }_{A}$ and E. The rate functions corresponding to each of the four regimes for 1A01 (${A}_{{{\mbox{I}}}}^{+},\,{A}_{{{{{{\rm{I}}}}}}}^{-},\,{A}_{{{\mbox{II}}}},\,{A}_{{{\mbox{III}}}}$) depend on both ${\sigma }_{A}$ and E, while the three regimes for 3B05 (${B}_{{{\mbox{I}}}},\,{B}_{{{\mbox{II}}}},\,{B}_{{{\mbox{III}}}}$) depend on E. These regimes are separated by the vertical red lines and horizontal dashed magenta line; see Supplementary Note 3 for a detailed description. In the schematic, black arrows with solid and dashed lines indicate effective and ineffective interactions in each regime. Red arrows indicate the change in the acetate concentration E, and the magenta arrows indicate the change of ${\sigma }_{A}$. Thick purple arrows indicate the crucial cross-feeding of acetate-induced metabolites allowing 3B05 to grow during acetate stress (and hence reduce the acetate concentration in the medium). **b** and **c** show numerical results of the model in (**a**) for the density of live 1A01 and 3B05 cells (top) and the concentration of GlcNAc, acetate, and acetate-induced metabolites (bottom) for the 24-h growth-dilution cycles with 1:1 initial species ratio. **b** Numerical results of the model during the stable cycle, which leads to two coordinated paths, ${A}_{{{\mbox{I}}}}^{+}\to \,{A}_{{{{{{\rm{I}}}}}}}^{-}\to {A}_{{{\mbox{II}}}}\to {A}_{{{\mbox{I}}}}^{+}$ for 1A01 (solid line) and ${B}_{{{\mbox{I}}}}\to \,{B}_{{{\mbox{II}}}}\to \,{B}_{{{\mbox{I}}}}$ for 3B05 (dashed line) over time. **c** Numerical results of the model at the end of each 24-h cycle.

To understand why the cross-feeding of pyruvate/lactate was able to rescue the co-culture after several growth-dilution cycles, we developed a mathematical model of acid-induced cross-feeding under growth-dilution dynamics (Fig. 6a, Supplementary Note 3). Quantitative account of the observed dynamical features by the model required not only the incorporation of strain characteristics obtained in monocultures as described above, including the excretion and uptake of the supplements as captured in Fig. 5, but also the lag of 1A01 and the growth of 3B05 during the first 6 h of the stable cycle (Fig. 4a). Additional experiments were performed to recreate this lag phase using 1A01 monoculture (Supp. Fig. 8a, b), to show that during this period, 1A01 cells continued to convert GlcNAc almost completely into acetate, pyruvate, and lactate and excreting them (Supp. Fig. 8c), likely providing for the growth of 3B05 in the initial phase of the stable cycle, despite the lack of acetate stress after being diluted into fresh culture. The large amount of acetate excreted indicates a bottleneck in the entry to TCA cycle, while supplement of certain metabolites related to the TCA cycle relieved the lag (Supp. Fig. 8d, e). The data thus suggest the origin of the lag phase to be the depletion of these and possibly other metabolites while they experienced acetate stress. This effect is described in our model as a memory effect by 1A01 cell upon encountering acetate stress. The resulting full model has most parameters fixed by our data, with minimal tuning only for kinetic processes inaccessible experimentally; see Supplementary Note 3 for a full description. The model was able to capture the stable-cycle dynamics quantitatively, including the timing and magnitude of the major metabolites around the acetate peak and the densities of the two species; compare the model output in Fig. 6b with the measurements in Fig. 4a, b, e. The model also captured the approach of the co-culture to the stable cycle, quantitatively reproducing the strain abundances and GlcNAc/acetate concentrations at the end of each cycle; compare the model output Fig. 6c to the observed data in Figs. 3b, d. Detailed accounts of stable-cycle dynamics and the approach to stable cycle are given in Supplementary Note 4.

Moreover, the model can be used to depict the details of how the co-culture organizes itself dynamically through each growth-dilution cycle to the stable cycle, e.g., for different initial ratio of the two species. At 3:1 initial ratio (of 3B05 to 1A01), the stable cycle is predicted to be reached within a single cycle instead of 3 cycles for 1:1 initial ratio, but with the same stable-cycle characteristics; see Supp. Fig. 9a, b. These two predictions about the dynamics with 3:1 initial ratio are verified in Supp. Fig. 9c, d. These results support the general notion that features of the stable cycle are properties of the community, independent of the initial condition and transient dynamics.

### Physiological basis for acid-induced cross-feeding

**Fig. 7:**
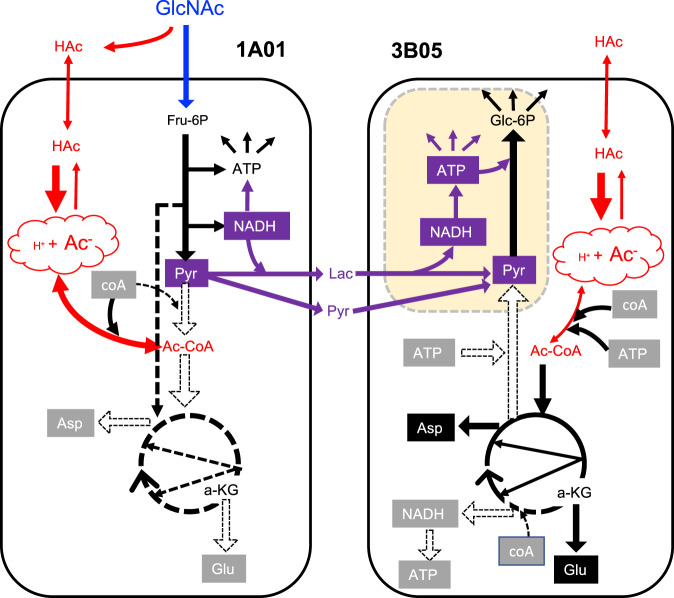
Metabolic model of acid-induced cross-feeding between 1A01 and 3B05. Schematic sketch indicating the key carbon fluxes in 1A01 and 3B05 during acetic acid stress. Metabolites in gray boxes are depleted, metabolites in red are related to acetate, and metabolites in purple (boxes or otherwise) are cross-fed from 1A01 to 3B05. Yellow box indicates 3B05 dynamics during recovery due to cross-feeding. Dashed arrows indicate reactions with negligible flux. The abbreviations are as follows: *N*-acetyl-glucosamine (GlcNAc), acetic acid (HAc), fructose-6-phosphate (Fru-6P), pyruvate (Pyr), lactate (Lac), coenzyme A (coA), acetyl-coA (Ac-coA), α-ketoglutarate (a-KG), aspartate (Asp), and glutamate (Glu). Acetic acid (HAc) is in equilibrium with the anion species, acetate (Ac^-^), with the ratio of the two concentrations governed by the pH, i.e., $\left[{{{{{\rm{A}}}}}}{{{{{{\rm{c}}}}}}}^{-}\right]=\left[{{{{{\rm{HAc}}}}}}\right]\times {10}^{{{{{{\rm{pH}}}}}}-{{{{{{\rm{pK}}}}}}}_{{{{{{\rm{a}}}}}}}}$ where ${{{{{\rm{p}}}}}}{{{{{{\rm{K}}}}}}}_{{{{{{\rm{a}}}}}}}\approx 4.75$. Because HAc is a small, neutral molecule, it is permeable through the cell membrane. The ratio of the intracellular and extracellular acetate concentrations is given by refs. ^44,45,53^ $\frac{{\left[{{{{{\rm{A}}}}}}{{{{{{\rm{c}}}}}}}^{-}\right]}_{{{{{{\rm{int}}}}}}}}{{\left[{{{{{\rm{A}}}}}}{{{{{{\rm{c}}}}}}}^{-}\right]}_{{{{{{\rm{ext}}}}}}}}={10}^{{{{{{{\rm{pH}}}}}}}_{{{{{{\rm{int}}}}}}}-{{{{{{\rm{pH}}}}}}}_{{{{{{\rm{ext}}}}}}}}$ assuming the pK_a_ does not change significantly in the cytoplasm. If the medium pH drops to ~5, and assuming internal pH is maintained at ~7, then 3 mM of acetate in the medium would result in ~300 mM in cells, on the order of the sum of the concentrations of all endogenous metabolites^75^. Based on detailed quantitative studies in *E. coli*, this obligatory flooding of the cytoplasm by acetate has two important physiological consequences: First, the high intracellular acetate pool completely ties up coenzyme A, dropping the free coA pool virtually to zero. Second, osmotic balance forces bacteria to adapt to the very high acetate pool by reducing the pools of many endogenous metabolites, particularly TCA intermediates and related amino acids such as glutamate and aspartate, to keep the total metabolite concentration (including acetate) roughly equal to that imposed by external osmolarity^53^. For sugar eaters like 1A01 growing on glycolytic substrates, a drop in the coA pool leads to the accumulation of pyruvate and drop in carbon influx, which in turn leads to a drop in the anapleurotic flux (dashed vertical black arrow in the left panel) and hence reduced growth. The lack of TCA intermediates would further limit the use of the high Ac-coA pool for respiration. Then, glycolytic flux must be increased, with concomitant increase in pyruvate excretion, to supply the cell’s energy needs; see left panel. Not much is known about the response of acid-consuming bacteria such as 3B05 to acetic acid stress. Given a high Ac-coA pool, most TCA intermediates can be readily generated in principle via the glyoxylate shunt. However, energy-generating reactions that produce NADH or NADPH (with proton as a by-product) tend to have optimal activities at elevated pH^76–78^; even enzymes reducing the quinone pools have been reported to exhibit reduced activities at reduced pH^79^. Furthermore, we expect the lack of coA to limit the synthesis of succinyl-coA. These effects all work towards limiting the flux of Ac-coA towards generating energy, which is very demanding for growth on acetate^59^. We expect this limitation in energy biogenesis to affect the flux of gluconeogenesis (open vertical arrow in the right panel), which is needed to generate glycolytic intermediates (pyr, pep, Fru-6P, etc.), en route to synthesizing approximately half of the biomass components. We hypothesize this bottleneck in the conversion of malate/oaa to pyr/pep due to energy limitation to be the direct cause of growth arrest for 3B05 during acetate stress; see right panel above. This hypothesis is supported by the observation of immediate and prolonged excretion of glutamate and aspartate upon exposing 3B05 to acetate stress (Supp. Fig. 10b), since this excretion indicates that TCA intermediates produced from acetate have nowhere to go. This excretion eventually stops over time, indicating a new state of 3B05 which does not take up acetate, presumably due to the accumulation/depletion of metabolites associated with the inability to assimilate acetate into biomass. The recovery of growth in response to the addition of pyruvate and lactate (Fig. 5a–c) further supports the gluconeogenesis bottleneck hypothesis (yellow box in the right panel), with the specifics of the bottleneck suggested by the detailed kinetics of growth recovery (Supp. Fig. 11). Together, we propose that 1A01 and 3B05 form a complementary metabolic partnership under acetate stress: 1A01 cannot move carbon past pyruvate and thus cannot fill the TCA intermediates. (Since 1A01 does not grow on acetate, it is presumably incapable of supplying TCA intermediates from Ac-coA alone.) Because it takes in sugar but does not grow, it has an excess of energy and carbon in the form of lactate and pyruvate. On the flip side, 3B05 has difficulty generating energy and supplying glycolytic intermediates via gluconeogenesis. Lactate and pyruvate from 1A01 relieve the growth bottleneck of 3B05, allowing it to resume growth and thereby consume acetate, the source of stress. While we have emphasized metabolic interactions in this model, we note that gene regulation would likely also play important roles during the growth recovery process as described in recent studies^53,80^.

To understand the origin of the positive interaction between 1A01 and 3B05 beyond acetate cross-feeding, we turn to the basic physiological problem faced by bacterial cells under acetate stress^44,45,53^. As explained in Fig. 7, the presence of a few mM of acetate at low external pH (~5) leads inevitably to the accumulation of a very high concentration of acetate in the cytoplasm with moderately reduced cytoplasmic pH and drastic decreases in the concentrations of endogenous metabolites. The remodeling of the metabolome has several important consequences on bacterial physiology: Based on results from a recent metabolomic study of *E. coli*^53^, we hypothesize that for bacteria growing on glycolytic substrates (such as 1A01 on GlcNAc), respiration becomes limited under acetate stress due to the depletion of TCA intermediates, and that these cells increase glycolytic flux for energy biogenesis. The lack of free coA (shifted mostly to acetyl-coA by mass action due to the high internal acetate concentration) then would force the glycolytic flux to be excreted as pyruvate. This scenario, depicted on the left side of Fig. 7 for 1A01, is supported by the substantial depletion of internal glutamate and aspartate, two amino acids reversibly connected to TCA intermediates, under acetate stress (open bars, Supp. Fig. 10a). This model rationalizes the continual consumption of GlcNAc, along with a nearly equal-molar excretion of pyruvate and lactate, for growth-arrested 1A01 cells under acetate stress (Fig. 5d–f, Supp. Fig. 7b). (Co-excretion of lactate likely resulted from the additional need to release a portion of the NADH generated from glycolysis; see Fig. 7.) The extreme (~10×) depletion of aspartate during acetate stress (Supp. Fig. 10a) also supports the effectiveness of aspartate supplement on growth recovery after stress (open squares, Supp. Fig. 8d).

Much less is known about the effect of acetate stress on acid-consumers such as 3B05, even though the drastic increase of acetate concentration in the cytoplasm and the accompanying remodeling of the metabolome discussed above are likely agnostic to organismal identity. Our data show that the glutamate and aspartate pools of 3B05 were similarly reduced as in 1A01 after experiencing acetate stress for a period of time (filled bars, Supp. Fig. 10a). However, immediately upon exposure to acetate stress, both glutamate and aspartate were excreted by 3B05 cells, and this persisted for over an hour (Supp. Fig. 10b), suggesting a surplus in the pools of TCA intermediates (which glutamate and aspartate are reversibly connected to) for some time after the onset of acetate stress. Indeed, unlike 1A01 which has limited flux towards TCA intermediates, 3B05 grows on acetate and can in principle fill most of the TCA intermediates from acetyl-coA using the glyoxylate shunt even during acetate stress (right panel, Fig. 7). However, we expect respiration of acetyl-coA by the TCA cycle to be affected significantly by acetate stress since the coA pool would be severely limiting during acetate stress. Additionally, as acidic conditions generally impede the reduction of oxidized electron-carriers (e.g., NAD^+^), it becomes more difficult to generate bio-available reducing power at lower pH. Given the large energy demand for growth on acetate^59^, we hypothesize that acetate-stressed 3B05 cells would have an acute energy shortage. We expect this shortage to be manifested in a limitation in gluconeogenesis^60^, i.e., the conversion of TCA intermediates into glycolytic intermediates such as pyruvate (dashed upward arrow, Fig. 7). A bottleneck in gluconeogenesis would also rationalize the recovery of 3B05 growth by the supplement of pyruvate and lactate (Fig. 5a, b), which provides the product of gluconeogenesis (yellow box, Fig. 7). However, the detailed kinetics of the recovery is more complex; see Supp. Fig. 11.

The scenarios described above and depicted in Fig. 7 for the metabolisms of 1A01 and 3B05 alone during acetate stress immediately suggest a mechanism of metabolic synergy between 1A01 and 3B05 in a co-culture: 1A01 extensively converts GlcNAc into acetate, pyruvate, and lactate even after it is growth-arrested due to self-acidification, while 3B05 uses pyruvate and lactate excreted by 1A01 to supplement its growth on acetate. This allows 3B05 to overcome its limited flux of gluconeogenesis, so that it can continue to consume and grow on acetate, eventually depleting acetate, the source of stress, for both species.

### Similar metabolic complementarity between unrelated bacteria

**Fig. 8:**
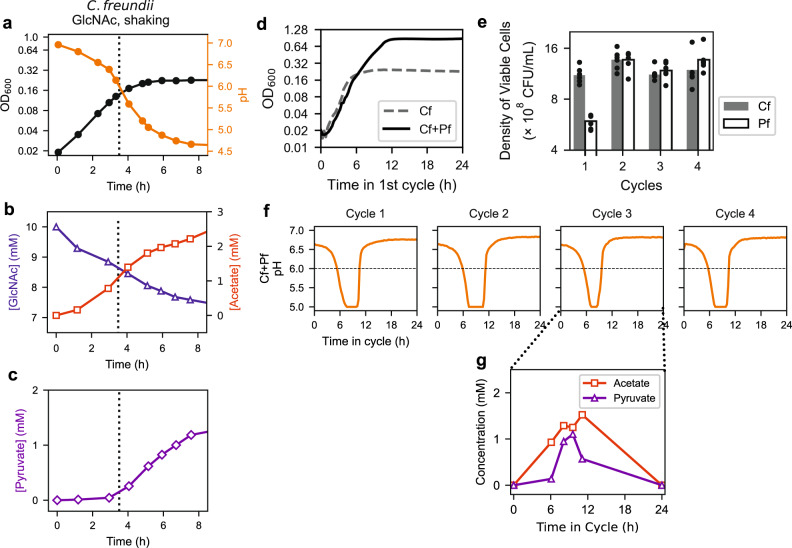
Acid-induced cross-feeding between soil bacteria growing on GlcNAc. Monoculture of *Citrobacter freundii* (Cf) or co-culture of Cf and *Pseudomonas fluorescens* (Pf) was grown in 10 mM GlcNAc as the sole carbon and nitrogen sources in weak phosphate-based buffer and with shaking; see “Methods”. **a**–**c** show data from monoculture growth of Cf with (**a**) OD and pH, (**b**) consumption of GlcNAc and excretion of acetate, and (**c**) excretion of pyruvate. **d**–**g** show data from 24-h growth-dilution cycles of Cf+Pf co-culture in the same medium with 100× dilution. The co-culture was started from a 1:1 mixture of exponentially growing cells of each species, each at an OD_600_ of 0.01. **d** Growth curve of the co-culture over the first cycle is shown as the solid black line. Growth curve of Cf monoculture (dashed gray line, same as the black curve in (**a**)) is shown for comparison. **e** The viable counts of Cf and Pf cells at the end of each cycle, with Cf and Pf colonies distinguished by their sizes. The data from six biological replicates are shown as filled black circles for each cycle. **f** pH dynamics was monitored continuously throughout the first four cycles; see Supplementary Fig. 12. The horizontal dashed black line indicates the pH below which acid-induced excretion was observed for the Cf monoculture (shown as vertical dotted lines in (**a**)–(**c**)). **g** Metabolites measured in the medium at selected times during Cycle 3. Purple triangles indicate the concentration of pyruvate and the red squares indicate the concentration of acetate.

As the above mechanism of collaborative resistance against acetate stress relies mostly just on the depletion of TCA intermediates upon the accumulation of acetate in the cytoplasm, and the latter is a general consequence of physiochemistry under acetate stress^44,45,53^, we expect it to be applicable generally across co-cultures involving bacteria with complementary metabolic types, i.e., glycolytically oriented sugar consumers and gluconeogenically oriented acid consumers. To test the predicted generality of the scheme of acid-induced cross-feeding depicted in Fig. 7, we selected *E. coli* along with three bacterial species from a previously studied soil bacterial consortium^18,20,61^, and subjected them to growth-dilution cycles. We paired a species from the Enterobacteriaceae family, which prefers growing on sugars while excreting acetate, with a species from the Pseudomonadaceae family, which prefers growing on organic acids including acetate^20,62^. When *Citrobacter freundii* from Enterobacteriaceae was grown in GlcNAc alone with a weak phosphate-based buffer, the monoculture stopped growing at a low OD when pH dropped below 6 (vertical dashed line, Fig. 8a). Analyzing the spent media of the *C. freundii* monoculture, we found the accumulation of acetate and pyruvate, with the amount of acetate increasing above ~1.5 mM as pH dropped below 6 (Fig. 8b, c). Adding *Pseudomonas fluorescens* to the culture extended the saturating OD by 3–4 fold (compare solid and dashed line, Fig. 8d), suggesting the possibility of acid-induced cross-feeding in the co-culture. To test the occurrence of the latter, we first confirmed that the co-culture maintained coexistence over several 24-h growth-dilution cycles with comparable counts from each species (Fig. 8e). Using a pH-sensitive dye to continuously monitor the pH dynamics in co-cultures (Supp. Fig. 12), we found that the pH dropped below 6 for several hours in the middle of each growth-dilution cycle, before recovering to the starting pH (Fig. 8f). Analysis of the spent medium revealed the accumulation of pyruvate in addition to acetate, peaking during the trough of the pH dip (Fig. 8g). These observations are highly analogous to the dynamics exhibited by the 1A01-3B05 co-culture growing on GlcNAc (Fig. 4), despite the very different characteristics of these species pairs.

**Fig. 9:**
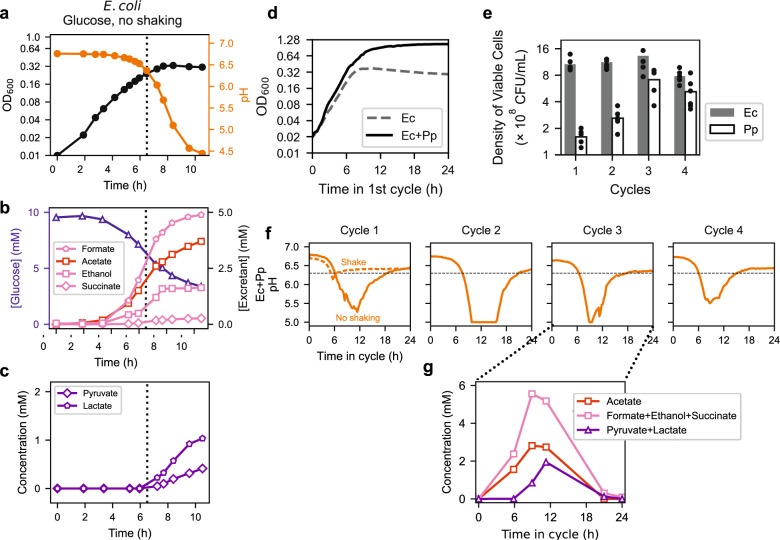
Acid-induced cross-feeding arising from transient anaerobic growth condition. Monoculture of *E. coli* (Ec) or co-culture of Ec and *Pseudomonas putida* (Pp) was grown in 10 mM glucose as the sole carbon source, in a weak phosphate-based buffer and kept without shaking; see “Methods”. **a**–**c** show data from monoculture growth of Ec with (**a**) OD and pH, (**b**) consumption of glucose and excretion of the suite of fermentation products normally associated with anaerobic growth^63^, acetate, succinate, formate, and ethanol, and (**c**) excretion of pyruvate and lactate. **d**–**g** show data from 24-h growth-dilution cycles of Ec+Pp co-culture in the same medium with 100× dilution. The co-culture was started from a 1:1 mixture of exponentially growing cells of each species, each at an OD_600_ of 0.01. **d** Growth curve of the co-culture over the first cycle is shown as the solid black line. The growth curve of Ec monoculture (dashed gray line, same as the black curve in panel (a)) is shown for comparison. **e** Viable counts of Ec and Pp cells at the end of each cycle, with Ec and Pp colonies distinguished by their sizes. The data from six biological replicates are shown as filled black circles for each cycle. **f** pH of the co-culture throughout the first four cycles is shown as the solid orange line. The dashed orange line in Cycle 1 indicates the pH of the coculture when shaken. The horizontal dashed black line indicates the pH below which acid-induced excretion was observed under identical growth conditions for the Ec monoculture (shown as vertical dotted lines in (**a**)–(**c**)). **g** Metabolites measured in the medium at selected times during Cycle 3. The sum of the concentrations of succinate, formate, and ethanol is shown as pink squares. Sum of the concentrations of pyruvate and lactate, which are not seen as fermentation by-products for anaerobically grown *E. coli* in strong buffer^63^ and hence interpreted as induced by acetate stress, is shown as purple triangles.

GlcNAc is a unique sugar with an extra acetyl group, whose catabolism leads to a steady excretion of acetate under aerobic growth (Supp. Fig. 2). To see whether acid-induced cross-feeding between pairs of growing species established here may be applicable to other means of acetate accumulation, we also examined the effect of self-acidification through poor aeration by culturing with exposure to air but not shaking, as was done in a number of recent studies^18,20,61^; see “Methods”. Here, we chose *E. coli* as the sugar-consuming acid excreter and *Pseudomonas putida* as the acid eater. Growing *E. coli* alone in glucose minimal medium with a weak phosphate-based buffer, we again found the monoculture to stop growing as pH dropped (Fig. 9a). The medium accumulated acetate, succinate, formate, and ethanol (Fig. 9b), the canonical fermentation products excreted by *E. coli* during anaerobic growth^63^, with lactate and pyruvate accumulating as pH dropped and growth was arrested (vertical dashed line, Fig. 9c).

Addition of *P. putida* again substantially extended the growth of the co-culture, suggesting cross-feeding (Fig. 9d). The two species coexisted in 24-h growth-dilution cycles (Fig. 9e), and pH dynamics again revealed the repeated dip for several hours in the middle of each cycle (Fig. 9f). Measurement of the co-culture media during the third cycle (Fig. 9g) showed the buildup and depletion of acetate (red squares), the remaining anaerobic excretants (pink squares), along with the acid-induced excretion (purple triangles). To confirm the role of oxygen deprivation in the observed phenomenon, we repeated the experiment with the co-culture shaken throughout the cycle: the sharp dip in pH disappeared in this case (dashed orange line, Fig. 9f, Cycle 1).

## Discussion

Weak organic acids are excreted by fast-growing sugar-consuming bacteria in many environments^34–42^. While these weak organic acids serve as natural growth substrate for a variety of acid-eating bacteria, typically the acid eaters grow more slowly than the sugar eaters and this mismatch of growth rates inevitably leads to a rapid buildup of the excreted acids as we found in a co-culture of the marine bacteria 1A01 and 3B05. This acid buildup would crash the pH once the buffer capacity of the medium is exceeded, putting the community of bacteria under acid stress. Because the acid eaters are not capable of growing on acid at low pH (Figs. 2e, 5a), a puzzle arises in how the co-culture is able to remove the acid and restore growth under repeated growth-dilution cycles (Fig. 3b, d). Using detailed, quantitative analysis, we revealed a hidden layer of metabolic collaboration that occurs in a co-culture of 1A01 and 3B05 during acid stress. As depicted in the metabolic model of Fig. 7, a positive interaction is realized whereby acid-induced excretion of pyruvate and lactate by 1A01 helped 3B05 to grow on the excreted acetic acid, hence detoxifying the environment for both species. Despite the complexity of the metabolic interactions, a simple dynamical model with constrained parameters was sufficient to capture the bulk of the observed dynamics (Fig. 6).

**Fig. 10:**
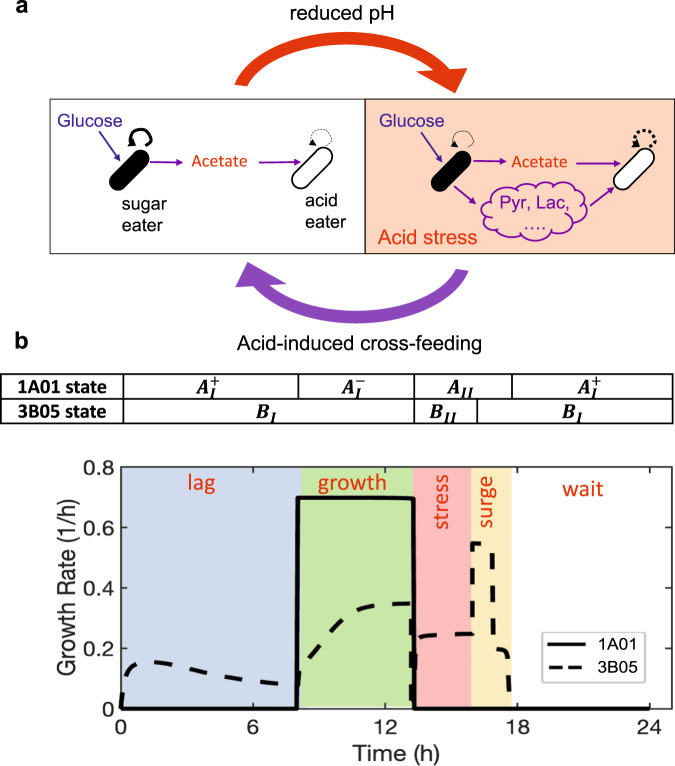
Different phases of the community dynamics. **a** Cartoon of acid-induced cross-feeding. During growth, weak acids are excreted by sugar eaters for a variety of reasons. As long as the excreted flux exceeds that of the consumption by “acid eaters” (left panel), the concentration of the excreted acid will accumulate in the medium, eventually reducing the medium pH when the accumulated acid exceeds the buffer capacity of the medium. The reduced pH results in acid stress that inhibits the growth of *both* species. During the stress, acid-induced cross-feeding enables the acid eater to remove the accumulated acid for both types of species (right panel). **b** Instantaneous growth rate of 1A01 (solid line) and 3B05 (dashed line) through a stable cycle according to the model (Fig. 6a, Supplementary Note 3). The abrupt changes in growth rates are defined by a number of phases of the co-culture, indicated by the colored bands. The latter arose due to a combination of the physiological states each species goes through during the cycle, as indicated by the table above the plot; the states of the individual species, ${A}_{I}^{+}$, ${B}_{{II}}$, etc. are defined in Fig. 6a and elaborated in Supplementary Note 3.

Rather than being an interaction specific to marine isolates, acid-induced cross-feeding appears to be a collaborative mechanism that occurs generally between complementary cell types—glycolytically-oriented, sugar-consuming acid excreters and gluconeogenically-oriented acid eaters (Fig. 10a). Recent work suggests that intrinsic limitations on the directionality of carbon metabolism force species to pick whether to excel at glycolytic or gluconeogenic metabolism^62^. Thus acid-induced cross-feeding is a positive interaction that arises not specifically for this purpose; rather it occurs as a by-product of the natural division of copiotrophic, heterotrophic bacteria into glycolytically and gluconeogenically oriented modes of metabolism. Here we showed acid-induced cross-feeding between *Pseudomonas* species from soil isolates and Enterobacteriaceae such as *C. freundii* and *E. coli* in addition to *Vibrio* sp. 1A01 and *Neptunomonas* sp. 3B05. Recent work suggests that *Staphylococcus aureus* and *Pseudomonas aeruginosa* likely have the same metabolic complementarity, and acid-induced cross-feeding may also promote the coexistence of these species during infection^64^.

Our data provide a physiological basis for the general idea of microbial diversity promoted by extensive cross-feeding among free-living bacteria. For each pair of co-culture we investigated, mM/OD levels of valuable metabolites were excreted in addition to acetate, allowing substantial growth by acid eaters on the excreted substrates. Sustained excretion at this level was not a minor leakage by stressed cells. In the case of 1A01, excretion was sustained by non-growing cells which actively took up GlcNAc from the medium and converted them almost completely into pyruvate and lactate (Fig. 5d–f). We suggest two benefits for such extensive excretion by 1A01: An immediate benefit is that, due to limited respiration in acetate-stressed cells^53^, glycolysis is an effective way for 1A01 to generate energy for its maintenance even when it is inhibited from growing (Fig. 7). Another is that 1A01 would die rapidly over the course of a day (Supp. Fig. 4) if it is left under acetate stress without rescue by 3B05, and the latter occurs only in the presence of large amounts of pyruvate and lactate excreted by 1A01. In all of the cases studied here, stress was a pre-requisite before excretion of metabolites other than acetate took place. This picture—in which stressed cells extensively excreted metabolites while not growing—challenges the current theoretical picture which assumes cells grow and share large amounts of metabolites continuously in growth-dilution scenarios^19,65^. In direct support of this multi-stage cross-feeding picture, coexistence of 1A01 and 3B05, which occurred in 24-h growth-dilution cycles featuring extended growth arrest, failed to establish in the same system under 6-h cycles that avoided growth arrest (Fig. 3e).

Our work suggests that niches for different species are created *out of* steady-state growth as gradients in stresses emerge^66–68^. Using our model which quantitatively captured co-culture dynamics (Fig. 6, Supp. Fig. 9), we plotted in Fig. 10b the growth rate of 1A01 and 3B05 throughout the duration of the stable cycle: The plot shows different species dominating in different phases of the cycle (indicated by the colored bands). The occurrence of these phases of differential dominance is a key ingredient for the maintenance of both species in growth-dilution cycles, as already suggested in early studies of simple growth-dilution dynamics^55,69^. As these phases resulted from interactions of different physiological states of the two species (indicated by the table at the top of Fig. 10b, with the states depicted in Fig. 6a), they should be even more relevant in more complex communities involving more species. Thus, species abundances and nutrient levels observed at the end of growth-dilution cycles in microbial ecology studies likely depend on the dynamics of the community throughout the cycle as established in simplified systems studied here, with metabolic and possibly other modes of interactions giving rise to distinct patterns of species dominance within different time windows of a cycle, in stark contrast to steady state models that have guided microbial ecology research for many decades^22,70^.

## Methods

### Strains

*Vibrio splendidus* sp. 1A01 and *Neptunomonas phycotrophica* sp. 3B05 were natural isolates obtained by ref. ^46^. In that work, ocean water collected near Woods Hole, MA, was mixed with chitin beads in the lab. 1A01 and 3B05 reached greater than 1% abundance on the surface of the beads at some point over the course of 6 days.

The additional strains used to test for acid-induced cross-feeding were *E. coli* NCM3722, *Citrobacter freundii* (ATCC# 8090), *Pseudomonas fluorescens* (ATCC# 13525), and *Pseudomonas putida* (ATCC# 12633).

### Growth media

#### Preparation of marine broth and LB agar plates

Marine broth medium was prepared by mixing 37.4 g of dried solid (Difco Marine Broth 2216) with ddH_2_O to 1-L. This solution was boiled for 1 min and allowed to cool before it was vacuum-filtered through a 0.22 μm filter. The solution was stored at room temperature. Marine broth (1.5%) agar plates were prepared by mixing together 2× marine broth medium (74.8 g/L) and 2× (30 g/L) autoclaved agar on a stir/hot plate. The temperature of this solution was maintained above 50 °C to prevent any agar solidification. Fifteen mL of the 1× marine broth/1.5% agar solution was added to a petri dish (Fisherbrand, 100 mm × 15 mm). Following solidification of the agar, the plates were stored in stacks face down in sealed bags at 4 °C. LB plates were prepared the same way, except 2× LB Broth (Miller, 50 g/L) medium was combined with 2× agar.

#### Preparation of “strongly buffered” HEPES minimal growth medium

We prepared a growth medium inspired by that used in the Marine Biological Laboratory’s Microbial Diversity Summer Course and MOPS medium used for the growth of enteric bacteria such as *E. coli*^71^. The benefits of this medium are 1) it is stable and supports steady-state growth of copiotrophic, heterotrophic marine bacteria to high densities, 2) it is easily made/purchased, and 3) it is clear and thus amenable to OD measurement of biomass.

We prepared the growth medium with HEPES as the buffer (“strongly buffered medium”) as follows. (i) Prepare 1 L of a 10× concentrate of by mixing the following: HEPES sodium salt, freshly prepared, 1.0 M, adjusted to pH 8.2 using 5 M HCl (400 mL); Tricine, freshly prepared, 1.0 M, adjusted to pH 7.4 with 5 M NaOH (40 mL); 1.0 M Na_2_SO_4_ (10 mL); trace metals (50 mL), a solution containing 7.6 mM FeSO_4_ · 7H_2_O, 0.48 mM H_3_BO_3_, 0.8 mM CoCl_2_·6H_2_O, 12 μM CuSO_4_, 0.5 mM MnCl_2_·4H_2_O, 0.5 mM ZnSO_4_·7H_2_O, 0.15 mM Na_2_MoO_4_·2H_2_O, 0.1 mM NiCl_2_ · 6H_2_O, 23 μM SeO_2_; and adding ddH_2_O to the mixture to 1 L. Filter sterilize this 10× concentrate by vacuum filtration through a 0.2 μm filter and store at −20 °C. (ii) Prepare 1 L of 4× concentrate of a simplified seawater (SW) mixture: 1.37 M NaCl, 59 mM MgCl_2_·6H_2_O, 4 mM CaCl_2_·2H_2_O, and 27 mM KCl. Filter sterilize and store at room temperature. (iii) Prepare a carbon source (i.e., 1 M sodium acetate), 1 M NH_4_Cl as the nitrogen source, and 0.5 M Na_2_HPO_4_ as the phosphorus source. Filter sterilize and store at room temperature (or −20 °C in the case of 0.4 M GlcNAc). (iv) To prepare the final growth medium, add the following to make 40 mL of 5 mM glucose medium, for example: 1) 25.32 mL of autoclaved ddH_2_O, 10 mL of 4× seawater, 0.2 mL of 1 M glucose, 0.4 mL of 1 M NH_4_Cl, 0.08 mL of 0.5 M Na_2_HPO_4_, 4 mL of 10× C-N-P-SW- concentrate. Vortex. This medium is stable at room temperature for at least a week. For medium with GlcNAc as the sole carbon source, no ammonium is provided unless otherwise indicated.

#### Preparation of bicarbonate (“weakly buffered”) minimal growth medium

We did not include tricine in the growth medium with sodium bicarbonate as the buffer since it affected buffering. Tricine was included in the HEPES buffered medium because iron would crash out upon storage at 4 °C due to the pH 8 of the medium. The bicarbonate buffered medium was stable (pH 7.5, iron remained solubilized) for at least 24 h at room temperature. To prepare 40 mL of GlcNAc growth medium with 2 mM bicarbonate carbonate as the buffer and no additional nitrogen source, we added the following: 28.38 mL of autoclaved ddH_2_O, 10 mL of 4× seawater, 0.5 mL of 0.4 M GlcNAc, 0.08 mL of 0.5 M Na_2_HPO_4_, 0.04 mL of 1 M Na_2_SO_4_, 0.2 mL of the trace metals mixture described above, and 0.8 mL of a 0.2 μm-filtered freshly-prepared solution of 0.1 M NaHCO_3_; then vortexed to mix. Note: we used this medium only after ~30 min to allow acid-base and bicarbonate equilibration with the atmosphere.

#### Preparation of phosphate-buffered medium

The base minimal medium for the soil strains was a 1× M9 medium^72^ (we used 10 mM NH_4_Cl instead of 18.7 mM) with 1× micronutrients and a carbon source. The 1000× micronutrient solution contained 20 mM FeSO_4_, 500 mM MgCl_2_, 1 mM MnCl_2_·4H_2_O, 1 mM CoCl_2_·6H_2_O, 1 mM ZnSO_4_·7H_2_O, 1 mM H_24_Mo_7_N_6_O_24_·4H_2_O, 1 mM NiSO_4_·6H_2_O, 1 mM CuSO_4_·5H_2_O, 1 mM SeO_2_, 1 mM H_3_BO_4_, and 50 mM CaCl_2_ dissolved in a 0.1 M HCl solution. For simplicity in the remainder of the methods, we call this base minimal medium “M9”.

For the *C. freundii*-*P. fluorescens* co-culture, we used M9 medium with the phosphate buffer component diluted 32× to give a buffer concentration similar to 2 mM bicarbonate. For the *E. coli*-*P. putida* co-culture we used M9 medium with the phosphate buffer component diluted 4×.

### Growth of monocultures

#### Batch monoculture growth

All cultures (except for those grown in the plate reader, see below) were grown in a water bath shaker at 27 °C with shaking at 250 rpm. We used this temperature for all growth experiments because the growth rate of 1A01 was highest at this temperature. We used this shaking frequency and the culture volumes specified below to ensure that oxygen availability was not limiting for OD_600_ < 1.5 (except for the *E. coli*-*P. putida* cocultures and *E. coli* monocultures, see “1A01 during and after exposure to acid stress” and “Growth of soil cocultures” of “Methods”). OD_600_ was measured using a Thermo Scientific GENESYS 30 Spectrophotometer.

Each growth experiment involved three steps: 1) a seed culture, 2) a preculture, and 3) an experimental culture. The seed culture was started by inoculating 2 mL of marine broth medium in a 16 mm × 125 mm test tube (borosilicate glass, Fisherbrand, Cat. No. 14-961-30) from a single colony on a marine broth/agar plate. Once the seed culture saturated (which took ~7 h for 1A01 and ~12 h for 3B05), the cells were washed and resuspended in 1× seawater to an OD_600_ of ~1 before being diluted into the experimental medium (3 mL in a 16 mm tube) for growth overnight, such that, by the following day, the preculture a) doubled $\ge$10 times and b) remained growing exponentially. While the preculture was still in exponential growth, we diluted the preculture into fresh experimental medium (8 mL in a 25 mm × 150 mm tube, prewarmed to 27 °C) to an OD_600_ of ~0.01. After another two doublings in the experimental culture, we took samples for various measurements, e.g., for the growth curve, spent media, etc. See “Sample collection” of “Methods” for details on sample collection. Whenever cells were washed with or transferred to another medium, it should be assumed that the medium was prewarmed to 27 °C unless otherwise indicated. Also, all wash steps were for 2 min × 7.5k × g unless otherwise indicated.

#### Growth on chitin

For the measurement of growth on chitin (Fig. 1a), we prepared 1A01 and 3B05 precultures in 10 mM GlcNAc (-N) and 60 mM acetate/10 mM NH_4_Cl HEPES minimal medium, respectively. We washed and resuspended the cells in C-N- HEPES minimal medium before adding 8 mL of HEPES minimal medium with 0.2% w/v chitin flakes and 10 mM NH_4_Cl (Millipore Sigma, C7170) to an OD_600_ of 0.05. No additional C source was provided. OD_600_ measurements were taken from well-mixed culture after allowing the visible chitin chips to sink for 4 min.

#### Measurement of pH-dependence of growth rate

For Fig. 2f, we precultured 1A01 and 3B05 each in 0.4% v/v glycerol HEPES minimal medium. We prepared a 96-well plate (Falcon, Product number 353072) containing 250 μL of 0.4% v/v glycerol minimal medium buffered by 10 mM MES. To vary the pH of the medium, we varied the ratio of the base and acid forms of MES and measured the pH using a Thermo Scientific Orion Star A221 pH meter. We allowed the plate to warm at 27 °C for 10 min in the plate reader before adding 1A01 or 3B05 to the wells.

To initiate growth in the plate, 2 mL of the preculture grown in HEPES medium with 0.4% glycerol was added to a well-containing medium in the 96-well plates described above such that the OD_600_ in the well (as measured by the plate reader) was ~0.0002. To allow for aeration of the cultures while avoiding evaporation of water and condensation on the lid, we attached the plate to its lid by lining the inside of the edges of the lid with high vacuum grease (Dow Corning). Note that we did not use grease on the corners of the lid to allow for aeration. The plate reader (Tecan Spark) was set to Orbital shaking with an amplitude of 2 mm and a frequency of 240 rpm. The OD_600_ was measured every 10 min. We measured the growth rate starting at OD_600_ ~ 0.03, so that the cells had ~7 doublings to acclimate to the pH. Following the cessation of growth, the volume of culture in the wells was spot checked for evaporation of water.

#### Measurement of 1A01 death rate in the absence of a nutrient

For Supp. Fig. 4d, we first grew a 1A01 preculture in GlcNAc (-N) HEPES medium to OD_600_ of 1. We sampled this preculture for plating as it grew exponentially. We then washed the cells twice and resuspended these cells in 18 mL of C-N- naturally buffered medium to an OD_600_ of ~1. We split this resuspension into two 25 mm tubes each with 9 mL of culture; in one of the tubes nothing was added so the pH remained at 7.5 and in the other tube we added 4 μL of 1 M acetic acid to a final concentration of 3.6 mM to lower the pH to 5.25. We then put both tubes back into the 27 °C water bath shaker and periodically sampled the cultures for OD_600_ and plating (Sampling of culture for OD_600_, pH, spent medium, and plating). Five hours after starting the cultures, we measured the pH of the two tubes again to confirm that the pH had not altered during the experiment.

#### 1A01 growth in manual “pH stat”

For Fig. 5d–f, we first grew 1A01 preculture in 10 mM GlcNAc (-N) HEPES medium. We washed the cells twice and resuspended the cells in 5 mM GlcNAc bicarbonate medium to an OD_600_ of 0.25 in a culture volume of 20 mL in a 250 mL flask. We allowed the culture to grow and acidify the medium through the excretion of acetate. When the pH reached ~5, we added small quantities of 0.1 M sodium bicarbonate (10–100 μL) to maintain the pH above 5. We sampled this culture for OD_600_, plating, and spent medium (“Sampling of culture for OD_600_, pH, spent medium, and plating”).

#### 3B05 in acid stress

For Fig. 5a–c, we first grew a 25 mL 3B05 preculture in 60 mM sodium acetate and 10 mM NH_4_Cl at pH 7.3 using 40 mM MOPS buffered growth medium. At this pH and concentration of total acetate, the concentration of acetic acid in the medium is ~0.2 mM; thus, 3B05 in this preculture had some exposure to acetic acid while still growing. We washed the cells twice and resuspended them into 1 mL of 2 mM HAc N-less bicarbonate buffered medium to an OD_600_ of 3.7. We then diluted these cells to an OD_600_ of ~0.1 to 2 × 25 mm tubes containing 8 mL of bicarbonate buffered media containing 4.5 mM of acetic acid and 1 mM of ammonium chloride. In one tube we added 1 M lactate, 0.5 M pyruvate, and 1 M glutamate to a final concentration of 1.5 mM, 1 mM, and 1 mM, respectively. We subsequently returned both tubes to the water bath shaker and periodically sampled the tubes for OD_600_ and spent medium (“Sampling of culture for OD_600_, pH, spent medium, and plating”).

We used a similar protocol to measure the metabolic response of 3B05 to acid stress (Fig. 5b, Supp. Fig. 10b). In brief, 3B05 acetate precultures in a strongly buffered medium were washed and resuspended in C-N- bicarbonate buffered medium. The resuspended cells were added to an OD_600_ of ~0.1 to a 25 mm tube containing 8 mL of bicarbonate buffered media to which 4.5 mM of acetic acid and 1 mM of ammonium chloride were added. Following the addition of 3B05 cells to the acidic growth medium, the cells and spent medium were sampled periodically.

We used a similar protocol to measure the use of pyruvate and lactate by 3B05 under acid stress (Supp. Fig. 11). In brief, exponentially growing 3B05 acetate precultures in strongly buffered medium were washed and resuspended in C-N- bicarbonate buffered medium. The resuspended cells were added to an OD_600_ of ~0.1 to a 50-mL conical tube containing 32 mL of bicarbonate buffered media to which 3.8 mM of acetic acid, 1 mM of ammonium chloride, and the requisite supplemented metabolites (described later) had already been added to the medium. The three supplemented metabolite conditions were 2.5 mM pyruvate, 2.5 mM lactate, and a combination of 1 mM pyruvate + 1.5 mM lactate, where the starting pH of the abiotic medium for each supplemented metabolite condition was similar: 5.02, 5.04, and 5.03, respectively. 30 mL of each biotic 32-mL culture was aliquoted into three, sperate 25 mm × 150 mm tubes (10 mL each) which served as biological triplicates. After the cultures were placed at 27 °C, shaking at 250 rpm, OD_600_ and pH were sampled throughout growth/deacidification.

#### 1A01 during and after exposure to acid stress

For Supp. Figs. 7 and 8, we first grew a 1A01 preculture in 5 mM GlcNAc (-N) bicarbonate buffered medium. We monitored the OD_600_ and pH of this culture as it acidified the medium during the course of growth due to acetate excretion. We sampled cells and spent medium for measuring internal amino acid concentrations and metabolomics (“HPLC method for measuring amino acid”, “High throughput mass spectrometry (FIA-TOF)”). The same method was used to grow *Citrobacter freundii* monoculture in Fig. 8a, b.

When the culture reached pH 5 (or other pH values as indicated in Supp. Fig. 8), we washed and resuspended the cells in fresh C-N- bicarbonate buffered medium. We added these cells to 5 mL of fresh 5 mM GlcNAc (-N) bicarbonate buffered medium such that the OD_600_ was 0.02 and returned the culture to the 27 °C shaker. We subsequently sampled this culture for OD_600_ and spent medium (“Sampling of culture for OD_600_, pH, spent medium, and plating”).

#### Monoculture growth of E. coli

For Fig. 9a–c, *E. coli* was grown exactly as the *E. coli*-*P. putida* co-culture (“Growth of soil cocultures”).

### Growth of cocultures

#### Coculture in HEPES buffer

As with monocultures, co-cultures of 1A01 and 3B05 were grown in a water bath shaker at 27 °C with shaking at 250 rpm. Whenever cells were washed with or added to growth medium to initiate a culture, the medium was prewarmed in the water bath shaker for at least 15 min.

For the growth-dilution experiments in Fig. 1c, we first grew 1A01 and 3B05 in marine broth for 12 hr, then washed and resuspended cells in 1× seawater. We then added each strain to 6 mL of prewarmed 5 mM GlcNAc (-N) HEPES minimal medium in a 20 mm × 150 mm tube such that the OD_600_ of each strain was 0.01 (total OD_600_ of 0.02). After 24 h, we added 150 μL of this culture to 5.85 mL of prewarmed 5 mM GlcNAc (-N) HEPES minimal medium in a 20 mm × 150 mm tube. We repeated this every 24 h until the conclusion of the experiment.

For later experiments involving coculture in HEPES buffer, we prepared 1A01 and 3B05 precultures in 10 mM GlcNAc (-N) HEPES and 60 mM acetate, 10 mM NH_4_Cl HEPES minimal media, respectively. We washed and resuspended each strain in 1× seawater before adding each strain to 6 mL of prewarmed 5 mM GlcNAc (-N) HEPES minimal medium in a 20 mm × 150 mm tube such that the OD_600_ of each strain was 0.01 (total OD_600_ of 0.02).

#### Coculture in weak (bicarbonate) buffer

We prepared precultures of 1A01 growing on 10 mM GlcNAc (-N) HEPES minimal medium and 3B05 growing on 60 mM acetate HEPES minimal medium. We washed and resuspended each of the strains into C-N- naturally buffered (2 mM bicarbonate) minimal medium. Then we added each of the strains in a 1:1 ratio to a total OD_600_ of 0.02 in 6 mL of prewarmed 5 mM GlcNAc (-N) naturally buffered minimal medium in a 20 mm × 150 mm tube. Both the C-N- and 5 mM GlcNAc (-N) naturally buffered minimal media were prepared ~30 min before use. Growth-dilution experiments were propagated into fresh 5 mM GlcNAc (-N) naturally buffered minimal medium as described above. Occasionally we observed aggregation/biofilm formation following the completion of a stable cycle. In those cases, we removed any spatial structure in the tube by vigorously pipetting up and down a 500 μL volume. The stable cycle for 1:1 and 3:1 3B05:1A01 starting ratios were measured following a cycle in which there was no aggregation by eye.

For the data indicated by the purple triangles in Supp. Fig. 5, we repeated the protocol described above using 1A01 and 3B05 colonies from frozen glycerol stocks of co-cultures collected at the end of a previous round of five growth-dilution cycles.

#### Mimic of the stable cycle in weak (bicarbonate) buffer

As the additional cross-feeding of internal metabolites in the stable cycle in naturally buffered medium occurs >12 h after the start of the cycle, we developed a way to observe this using a mimic. As both strains were growing exponentially for several hours before the growth arrest, we mimicked this exponential state of the stable cycle by starting a coculture using exponentially growing 1A01 and 3B05 monocultures (in 10 mM GlcNAc and 60 mM acetate/10 mM ammonium chloride, respectively), with the density of 3B05 adjusted to that found at ~8 h before the onset of growth arrest and the density of 1A01 kept small enough such that the following morning the coculture would reach the point 4 h before the onset of growth arrest.

#### Growth of soil cocultures

All batch culture growth of soil bacteria was performed at 27 °C, same as for 1A01 and 3B05. For the *C. freundii* (Cf)-*P. fluorescens* (Pf) coculture, we prepared pre-cultures of Cf growing on 10 mM GlcNAc M9 medium and of Pf growing on 60 mM acetate M9 medium. We washed and resuspended each of the strains into C- M9 medium with no phosphate buffer aside from 1 mM Na_2_HPO_4_. Then we added each of the strains in a 1:1 ratio to a total OD_600_ of 0.02 to prewarmed 10 mM GlcNAc M9 medium with 1/32× phosphate buffer to a total volume of 200 μL in a 96-well plate (Falcon, Product number 353072) with the lid greased as described in “Measurement of pH-dependence of growth rate”. (The OD_600_ measured on the Thermo Scientific GENESYS 30 Spectrophotometer was equivalent to absorption at 420 nm for 200 μL of culture in the Tecan Spark plate reader.) The plate reader was set to Orbital shaking with an amplitude of 2 mm and a frequency of 240 rpm. Every 15 min the absorption from 400 to 650 nm was measured, in 10 nm increments. After 24 h, 2 μL of the culture was added to 198 μL of fresh 10 mM GlcNAc M9 medium with 1/32× phosphate buffer.

For the *E. coli* (Ec)-*P. putida* (Pp) coculture, we prepared precultures of Ec growing on 10 mM Glucose M9 medium and of Pp growing on 60 mM acetate M9 medium. We washed and resuspended each of the strains into C- M9 medium with no phosphate buffer aside from 1 mM Na_2_HPO_4_. Then we added each of the strains in a 1:1 ratio to a total OD_600_ of 0.02 to prewarmed 10 mM GlcNAc M9 medium with 1/4× phosphate buffer to a total volume of 200 μL in a 96-well plate (Falcon, Product number 353072), with the lid greased as described in “Measurement of pH-dependence of growth rate”. The plate reader (Tecan Spark) was set to Orbital shaking with an amplitude of 2 mm and a frequency of 240 rpm for 2 min to mix the culture. Every 15 min the absorption from 400 to 650 nm was measured, in 10 nm increments. Between absorption measurements, the Tecan was set to “Wait,” i.e., no shaking. After 24 h, the plate was shaken for 2 min as at the start of the cycle to mix the culture, then 2 μL of culture was added to 198 μL of fresh 10 mM Glucose M9 medium with 1/4× phosphate buffer.

To monitor the pH, we added 2 μL of 0.04% bromocresol purple dye (Sigma, Product No. 114375) in a duplicate culture in a separate well at the start of the cycle. To calculate the change in the absorption spectrum of the dye, we 1) calculated the difference in absorption between the well with dye and the well without dye to remove cell background, 2) calculated the difference between the background-corrected absorption measured at 590 nm and that measured at 650 nm, and 3) calculated the pH corresponding to this value using a standard curve made using buffer solutions with known pH values (Supp. Fig. 12).

### Sample collection

#### OD_600_ for growth curve

For measuring the growth curves shown in Fig. 1, the OD_600_ was measured by taking the tube out of the shaker, taking out 200 μL, putting the tube back into the shaker, and then pipetting the 200 μL of culture into a quartz cuvette sitting in the spectrophotometer. The reading would stabilize within 5 s. The culture spent <10 s out of the shaker for each of these measurements.

#### Sampling of culture for OD_600_, pH, spent medium, and plating

When the culture was sampled for OD_600_, spent medium, and plating, the culture was taken out of the shaker, and ~700 μL was pipetted into an Eppendorf tube. The culture was then put back into the shaker within 30 s.

First, 10 mL of the culture in the Eppendorf tube was added to 990 μL of marine broth to start the dilutions for plating. Second, 200 μL of culture was used to measure the OD_600_. Third, the remaining culture in the tube was centrifuged to pellet the cells for 2 min at 7.5k × g. The supernatant (~500 μL) was then added to a Spin-X centrifuge tube containing a 0.22 μm filter (Corning Life Sciences, Costar 8169) and centrifuged for 1 min × 9.2k × *g*. The pH was measured on either the culture used for measuring OD_600_ or the filtered medium using a Thermo Scientific Orion Star A221 pH meter. During the two centrifuge steps for collecting spent medium, subsequent dilutions into marine broth were carried out for plating, as well as the spreading of cells on the plates (see Plating section for more details). The OD_600_ was measured within 1 min of collecting the culture, and the spent medium collection and pH measurement took ~6 min. Dilutions for plating were initiated ~5 min after culture collection and completed 10 min after that.

The spent medium was stored at −20 °C.

#### Intracellular metabolites

We followed the no-harvest protocol described by ref. ^73^ with some modifications.

To extract intracellular metabolites from a culture, 150 μL of culture was immediately (within 10 s) added to an Eppendorf tube containing a mixture of ice-cold 600 μL methanol and 30 μL of 50 μM a-amino-adipate (AAA, internal standard); the tube was vortexed for 5 s and placed into dry ice. Immediately (within 10 s), 200 μL of spent media was collected using centrifuge filtration (as described in “Sampling of culture for OD_600_, pH, spent medium, and plating”). 10 μL of AAA was added to this spent medium, the mixture vortexed for 5 s, and placed into dry ice. The culture was not used following these sample collections. Both the culture and spent medium samples were stored at −80 °C.

The lid of the Eppendorf containing the cell sample was opened, and the opening was covered with Parafilm, with 10–15 holes in the Parafilm made using a needle. The tube was then placed SpeedVac vacuum concentrator. The samples dried after ~3 h under vacuum. The resulting pellet was resuspended in 150 μL of 0.22 μm-filtered ddH_2_O; vortexed for 30 s; heated for 1 min at 37 °C; then vortexed again for 30 s. Cell debris was removed by centrifuge filter as described in “Sampling of culture for OD_600_, pH, spent medium, and plating” for the isolation of spent medium. 40 μL of this sample was used for HPLC measurement.

#### Cell pellets for 16S amplification and sequencing

For determining the ratio of the two species at the end of a growth-dilution cycle using 16S sequence amplification followed by Sanger sequencing, we first centrifuged 0.95 mL of the culture for 10 min × 7.5k × g, as recommended by the Qiagen DNeasy Blood and Tissue Kit protocol for Gram-negative bacteria. Following centrifugation, the spent medium was removed using a 1 mL pipette, with significant care taken to not dislodge the pellet. Because the pellet loosened within 30 s following centrifugation, only two pellets could be isolated per centrifugation to ensure the pellet composition resembled the culture composition. We note that 3B05 inefficiently pelleted as its density in the culture increased; as a result, its proportion in cocultures where its OD_600_ was >0.2 was underestimated by subsequent quantification by 16S amplification and sequencing. The pellets were stored at −80 °C.

#### Sampling of spent medium from 96-well plate

For measuring spent medium from the cultures grown in 96-well plate (“Monoculture growth of *E. coli*“ and “Growth of soil cocultures”), we initiated several duplicate cultures in the same plate so that each duplicate underwent the same growth dynamics. To sample a single timepoint of these dynamics, the entire 200 μL volume of a duplicated culture was harvested for the spent medium (Sampling of culture for OD_600_, pH, spent medium, and plating).

### Plating

As described in “Sampling of culture for OD_600_, pH, spent medium, and plating“, plating of 1A01 and 3B05 was initiated by adding 10 μL of the culture to 990 μL of marine broth (either at room temperature or 27 °C). This dilution was mixed by pipetting 500 μL up and down. Further dilutions were done in marine broth and by mixing the same way. 100 μL of the diluted culture was added onto marine broth/agar plates prewarmed to 27 °C. The culture was spread using autoclaved glass beads and dried in a PCR hood until no liquid was visible on the surface of the plate. We incubated the plate in an oven at 27 °C for at least 24 h. Plates with >100 and <300 colonies were counted by hand.

Plating of *E. coli*, *C. freundii*, *P. putida*, and *P. fluorescence* was done similarly as above, but using LB plates and cultured at 30 °C. Cf and Pf colonies from the Cf+Pf co-culture are distinguished by their size, with Pf colonies being significantly smaller after plating on LB for 24-h at 30 °C. Ec and Pp colonies from Ec+Pp cocultures are similarly distinguished by size.

### Assays for measuring metabolites

#### HPLC method for measuring carbohydrates and organic acids

We quantified the consumption of carbohydrates and excretion of organic acids in spent medium using HPLC. We added 120 μL of spent media to a vial. The chromatographic system was a Shimadzu LC-20AB connected to a Shimadzu RID-20A refractive index detector. The auto-injector delivered a 20 μL injection volume from the vials to a Rezex ROA-Organic acid H^+^ (8%) column (Phenomenex) kept at 40 °C. The solvent system was 0.01 M H_2_SO_4_ with a flow rate of 0.4 mL/min. We analyzed the data using homemade Python code. Absolute concentrations were obtained by comparing the peak areas obtained in a sample with those from standards with known concentrations.

#### HPLC method for measuring amino acids

The same LC system described above was used with a Gemini 5 mm C18 110 Å column 150 × 4.6 mm (Phenomenex) and fluorescence detection (RF-10AXL, Shimadzu). Using the LC’s auto-sampler, 10 μL of the sample was derivatized with an *o*-phthaldialdehyde (OPA) solution^73^ for fluorescence detection. A gradient elution for separating the derivatized amino acids was used with two solvents: solvent A was 90% sodium acetate, 9.5% methanol, and 0.5% THF set at pH 7.2; solvent B was 100% methanol. The gradient sequence was as follows: 0 to 10% of B over 6 min; 10% of B from 6 to 21.75 min; 10% to 80% of B from 21.75 to 22.5 min; 80% of B from 22.5 to 34.5 min; 80% to 0% of B from 34.5 to 35.25 min; 0% of B until 36 min (end of the run). This gradient could clearly separate aspartate, glutamate, asparagine, serine, and glutamine OPA derivatives.

The concentration of an amino acid (e.g., glutamate) in a sample was calculated by (1) calculating the ratio of the peak areas of 5 μM glutamate standard to 5 μM AAA standard; (2) calculating the ratio of the peak areas of glutamate to AAA in the sample; (3) dividing ratio (2) by ratio (1); and (4) multiplying (3) by the concentration of AAA in the sample.

To calculate the cellular amino acid content (in μmol/OD/mL), the concentration of the amino acid extracted from the “culture” sample (cells + spent media, see “Intracellular metabolites“) and from the spent media sample taken at the same time (“Intracellular metabolites”) were measured and calculated to units of μmol/mL. The intracellular amino acid content was then obtained as the difference between two quantities, further divided by the OD of the culture when the samples were harvested.

#### Enzymatic assay for ammonium concentration

We adapted the assay procedure for L-Glutamic Dehydrogenase from Sigma for measuring ammonia (ammonium) concentrations in the spent medium. We mixed the following reagents (kept ice cold until mixing):

- 700 μL of 0.1 M Tris buffer, pH 8.3
- 66.7 μL of sample (e.g., spent medium or NH_4_Cl standard)
- 33.3 μL of 0.225 M a-ketoglutarate, pH 7–9
- 16.7 μL of 7.5 mM NADPH

and incubated at 30 °C for 5 min. Then we added 16.7 μL of a 500× dilution of L-Glutamic Dehydrogenase (Millipore Sigma, G4387-1KU) diluted with the Enzyme Diluent indicated in the protocol, mixed by pipetting up and down, aliquoted 250 μL into three wells of a 96-well plate, and started measuring absorption at 365 nm in a plate reader. The change in absorption at 365 nm after 4 min varied linearly with known ammonium chloride concentrations between 0 and 1 mM. We averaged the results of three wells to get the results for a single spent media sample.

#### High throughput mass spectrometry (FIA-TOF)

Samples were prepared for untargeted metabolomics by diluting supernatants 1:20 in water. Samples were directly injected and measured using flow-injection time-of-flight mass spectrometry. Measurements were performed using a binary LC pump (Agilent Technologies) and an MPS2 autosampler (Gerstel) coupled to an Agilent 6520 time-of-flight mass spectrometer (Agilent Technologies). Measurements were performed in negative ionization mode, at 2 Ghz for an extended dynamic range, with an m/z (mass over charge ratio) range of 20–450. To reduce the matrix effects induced by high salt concentrations^58^, isocratic measurements were coupled to an Agilent Poroshell 120 EC-CN column (50 × 2.1 mm, 2.7 μm). Due to the poor retention of compounds on the column, the injection peak was treated as a flow-injection approach for downstream data analysis. The mobile phase consisted of 10 mM ammonium acetate pH 5.9, and the flow rate was 250 μL/min. 2 μL of sample was injected every 2.5 min. After every 30 injections, the column was washed for 5 min with a buffer that contains water (40%), isopropanol (30%), and acetonitrile (30%). Raw data were processed and analyzed with preprocessing raw mass spectrometry data functions contained in the bioinformatics toolbox of Matlab (The Mathworks, Natick)^57^. Ions were annotated with a tolerance of 0.005 Da against a compound library that is curated from BioCyc databases^74^, which contains metabolites predicted to be present in marine bacterial isolates. 957 ions were detected, of which 124 were annotated based on the curated compound library. If a single ion is matched with multiple isomeric or isobaric compounds in the compound library, the compound that participates in the largest number of enzymatic reactions based on the BioCyc database was chosen as the top annotations.

Metabolites were first filtered to contain only those that surpass the limit of detection for at least one timepoint over the course of measurement for each timepoint represented in Fig. 4f, and Supp. Fig. 7. This is defined as having a mean intensity that is greater than the mean intensity of a blank sample plus three times the standard deviation of the blank sample.

The data in Fig. 4f was plotted as follows. The scaled intensity for a single metabolite time course was calculated by taking the difference between the intensity and the intensity at the first time point and then dividing by the maximum such value for that time course. Metabolites for which the scaled intensity of the last time point is less than 0.5 are plotted in purple. Other detected metabolites are plotted in gray.

The metabolomic data in Supp. Fig. 7 was plotted by dividing the intensity by the maximum intensity for a single metabolite time course.

### Assays for measuring coexistence

#### 16S PCR and Sanger sequencing

We isolated the genomic DNA from a pellet obtained as described in “Cell pellets for 16S amplification and sequencing“ using the Qiagen DNeasy Blood and Tissue Kit, using the protocol suggested for Gram-negative bacteria. We amplified the 16S region using the following primers:

- 27F: AGAGTTTGATCMTGGCTCAG
- 1492R: TACGGYTACCTTGTTACGACTT

The components of the PCR reaction mix are listed in Supplementary Table 3, and the PCR cycling conditions are listed in Supplementary Table 4. The result of the PCR reaction was a 1506 nt product. We chose 54 °C as the annealing temperature because this was the lowest temperature at which neither species gave a non-specific second band at ~1 kb. A warning: The M residue in 27F is some mixture of A and C that varies between different oligo syntheses. For one such oligo, the non-specific band at ~1 kb would not go away even with higher annealing temperatures. Thus, either A or C results in more specific binding of the 27F primer for these two species.

For a single genomic sample, we pooled together two PCR reactions and purified the PCR product using a QIAquick PCR purification kit (Qiagen). We prepared a sample for Sanger sequencing (Genewiz) by mixing 30 ng of the purified PCR product with 6 pmol of the 27F primer in 15 mL. We fit the electropherograms using the CASEU package^48^ to get the fraction of each species’ 16S sequence in the mixture of amplicons. With high-quality electropherograms of 1A01 and 3B05, we routinely got an *R*^2^ > 0.9 on fits of mixtures of amplicons using the default settings in the package.

#### CFU count

We performed the plating procedure as described in “Plating”. The two strains in each co-culture are distinguished by their different colony sizes that could be detected by visual inspection; see Supplementary Fig. 3 for colonies 1A01 and 3B05.

### Simulations

Numerical simulations of the models described in Fig. 2g, h, Fig. 6b, c, Supp. Figs. 9a, b, 13, and 14 were performed using Python or Matlab.

### Reporting summary

Further information on research design is available in the Nature Portfolio Reporting Summary linked to this article.

## Supplementary information

Supplementary Information

Peer Review File

Reporting Summary

## Data Availability

Raw mass spectral data is deposited to massIVE and accessible with the accession code MSV000087136. Source data are provided with this paper.

## Source data

Source Data

## Code availability

The code used to model the dynamics of the co-culture based on the mathematical model presented in Supplementary Note 3 is available at https://github.com/avaneeshnarla/dynamic-metabolic.
