# Supplementary material for: Stress-induced metabolic exchanges between complementary bacterial types underly a dynamic mechanism of inter-species stress resistance
Source: Nat Commun. 2023 May 31;14:3165. doi: 10.1038/s41467-023-38913-8 (PMC10232422; doi:10.1038/s41467-023-38913-8)
Supplement: Supplementary file 1 — Supplementary Information [file 41467_2023_38913_MOESM1_ESM.pdf]

Stress-induced metabolic exchanges between complementary bacterial types underly a dynamic mechanism of inter-species stress resistance

Kapil Amarnath, Avaneesh V. Narla, Sammy Pontrelli, Jiajia Dong, Jack Reddan, Brian R. Taylor, Tolga Caglar, Julia Schwartzman, Uwe Sauer, Otto X. Cordero, Terence Hwa

Supplementary Information

Table of Contents:

Supplementary Tables 2

Supplementary Figures 6

Supplementary Note 1 23

Supplementary Note 2 25

Supplementary Note 3 26

Supplementary Note 4 32

Supplementary References 35

Supplementary Tables

strain	carbon & nitrogen sources	growth rate (h^{-1})	specific uptake of carbon substrate (mM/OD)	specific excretion of acetate (mM/OD)	specific excretion of ammonium (mM/OD)	specific uptake of ammonium (mM/OD)	viable cell density at OD_{600} = 1 (CFU/mL)
Vibrio splendidus sp. 1A01	GlcNAc	0.81±0.01	5.9±0.1	9.3±0.3	1.5±0.03	-	8.0±0.2 E8
	Glucose, Ammonium	0.83±0.04	5.7±0.3	2.3±0.2	-	4.1±0.2	
Neptumonas phycotrophica sp. 3B05	Acetate, Ammonium	0.43±0.01	32±2.4	-	-	4.1±0.2	1.3±0.2 E9
	Acetate, Ammonium, Lactate, Pyruvate, Glutamate	0.56±0.02	-	-	-	-	-
E. coli K-12 NCM3722	GlcNAc	0.57±0.03	5.1±0.4	10.5±0.4	2.1±0.3		
	Glucose, Ammonium	0.59±0.04	5.6±0.2	3.9±0.1	-		

Supplementary Table 1. Monoculture steady-state physiological parameters for *V. splendidus* sp. 1A01, *N. phycotrophica* sp. 3B05, and *E. coli* NCM3722. Note that the growth yield on a substrate is the inverse of the specific uptake of that substrate listed in the table, with biomass density given in unit of OD_{600}; the conversion of the latter to cell density is given by the last column. 1A01 and 3B05 were grown at 27°C in 0.35 M NaCl in the strongly buffered HEPES medium described in **Methods**; all parameters were the same in the weakly buffered medium with 2mM sodium bicarbonate, except for 1A01's GlcNAc growth rate, which was 0.72/h, and 3B05's growth rate on acetate, which was 0.35/h (**Fig. 4c**). *E. coli* NCM3722 was grown at 37°C in MOPS medium with 0.4 M NaCl. The standard error for each parameter is estimated from standard deviation of least squares fit of data.

Cross-fed Metabolites (Purple)	Remaining Metabolites (Grey)
Glycerate	2-hydroxyglutarate
1-amino-propan-2-one-3-phosphate	2-aceto-lactate
3-dehydroshikimate	Acetone
4-amino-butyrate	Dyhydroxyphenylglycol
Coumaraldehyde	2-methyl-maleate
4-hydroxybenzoate	Glycine
5-oxoproline	L-asparate-semialdehyde
Ribulose-5-phosphate	L-aspartate
Ascorbate	L-glutamate-semialdehyde
Glutamate	Acetyl-glutamate
Pyruvate	

Supplementary Table 2. Metabolites measured by untargeted metabolomics during the 'acetate peak' of the coculture. Fig. 4f of the main text shows plots of the metabolite dynamics measured by untargeted metabolomics of the spent medium during the 'acetate peak' of the stable cycle. The cross-fed (purple lines in **Fig. 4f**) and the remaining metabolites (grey lines) are listed above. The determination of whether a metabolite was cross-fed or not is discussed in **Methods**.

	Volume for 1 rxn
ddH_2O	20.8 µL
5x HF buffer	8.0 µL
dNTPs	0.8 µL
27F (3 µM)	4 µL
1492R (3 µM)	4 µL
Template (100 ng)	2 µL
Phusion polymerase	0.4 µL
Total	40 µL

Supplementary Table 3. Components and volumes of the 40 µL PCR reaction mix used for amplification of the 16S region. The entire amplification process is detailed in **Methods 8.1**.

**Step**	**Temperature**	**Time**
Initial denaturation	98°C	30 s
Amplification	98°C	30 s
(25 cycles)	54°C	30 s
	72°C	90 s
Final extension	72°C	10 min

Supplementary Table 4: PCR cycling conditions for the amplification of the 16S region using an Eppendorf Mastercycler epgradient. The entire amplification process is detailed in **Methods 8.1**.

Model for 3B05	$E \leq E_{B1}$	$E \geq E_{B1}$	$E \gg E_{B2}$
λ_B	$r_{B,E} f\left(\frac{E}{K_E}\right) + r_{B,M} f\left(\frac{M}{K_M}\right)$	$r_B^+ f\left(\frac{E}{K_E}\right) f\left(\frac{M}{K_M}\right) \theta(E_{B2} - E)$	$\rightarrow 0$
$\mu_{B,E}$	$r_{B,E} f\left(\frac{E}{K_E}\right) / Y_{B,E}$	$b r_B^+ f\left(\frac{E}{K_E}\right) f\left(\frac{M}{K_M}\right) \theta(E_{B2} - E) / Y_{B,E}$	$\rightarrow 0$
$\mu_{B,M}$	$r_{B,M} f\left(\frac{M}{K_M}\right) / Y_{B,M}$	$(1 - b) r_B^+ f\left(\frac{E}{K_E}\right) f\left(\frac{M}{K_M}\right) \theta(E_{B2} - E) / Y_{B,M}$	$\rightarrow 0$

Supplementary Table 5: Forms of the growth rate of 3B05, λ_B, and its uptake rate for acetate and pyruvate/lactate, $\mu_{B,E}$ and $\mu_{B,M}$, respectively.

Model for 1A01	$E \leq E_{A1}$		$E_{A1} \leq E < E_{A2}$	$E \geq E_{A2}$
	$\sigma_A > \sigma_A^c$	$\sigma_A \leq \sigma_A^c$		
λ_A	$r_A f(G/K_{A,G})$	0	0	$-\delta_A$
$\mu_{A,G}$	$r_A f(G/K_{A,G})/Y_{A,G}$	$\mu_{A,G}^{lag} f(G/K_{A,G})$	$\mu_{A,G}^{str} f(G/K_{A,G})$	0
$\mu_{A,E}$	$r_A f(G/K_{A,G})/Y_{A,E}$	$\mu_{A,E}^{lag} f(G/K_{A,G})$	$\mu_{A,E}^{str} f(G/K_{A,G})$	0
$\mu_{A,M}$	0	$\mu_{A,M}^{lag} f(G/K_{A,G})$	$\mu_{A,M}^{str} f(G/K_{A,G})$	0

Supplementary Table 6: Forms of the growth rate of 1A01, λ_A, its uptake rate for GlcNAc ($\mu_{A,G}$), and its excretion rate for acetate and pyruvate/lactate, $\mu_{A,E}$ and $\mu_{A,M}$, respectively.

Parameter	Value used	Description	Source
$\boldsymbol{r_A}$	0.7/h	Growth rate of 1A01 on GlcNAc	Supp. Table 1
$\boldsymbol{\mu_{A,M}^{str}}$	1.7 mM/OD/h	Rate of metabolite excretion by 1A01 during acetate stress	Fig. 5
$\boldsymbol{\mu_{A,G}^{str}}$	1.0 mM/OD/h	Rate of GlcNAc consumption by 1A01 during acetate stress	Fig. 5
$\boldsymbol{\mu_{A,E}^{str}}$	1.1 mM/OD/h	Rate of acetate excretion by 1A01 during acetate stress	Fig. 5
$\boldsymbol{\mu_{A,M}^{lag}}$	0.81 mM/OD/h	Rate of metabolite excretion by 1A01 during growth lag	Supp Fig. 8a-c
$\boldsymbol{\mu_{A,G}^{lag}}$	0.81 mM/OD/h	Rate of GlcNAc consumption by 1A01 during growth lag	Supp Fig. 8a-c
$\boldsymbol{\mu_{A,E}^{lag}}$	1.2 mM/OD/h	Rate of acetate excretion by 1A01 during growth lag	Supp. Fig. 8a-c
$\boldsymbol{\delta_A}$	0.5/h	Death rate of 1A01 at high acetate stress	Supp. Fig. 4d
$\boldsymbol{r_{B,M}}$	0.2/h	growth rate of 3B05 on acid-induced metabolites alone	difference of the two rates shown in Fig. 4c
$\boldsymbol{r_{B,E}}$	0.35/h	Maximal growth rate of 3B05 on acetate	Fig. 4c
$\boldsymbol{r_B^+}$	0.25/h	Maximal growth rate of 3B05 on acetate and acid-induced metabolites during acetate stress	see discussion in paragraph below Eq. (3.8).
$\boldsymbol{\sigma_A^{str}}$	0.5/mM/h	Proportionality constant for increase of internal variable of 1A01 during acetate stress	chosen to account for observed lag time in Fig. 4a
$\boldsymbol{\sigma_A^{lag}}$	0.1/h	Proportionality constant for decrease of internal variable of 1A01 during recovery from acetate stress	
$\boldsymbol{\sigma_c}$	0.1	Threshold value of internal variable above which growth does not take place for 1A01	
$\boldsymbol{E_{A1}}$	3 mM	Threshold acetate concentration for 1A01 to experience acetate stress and excrete acid-induced metabolites rapidly	Fig. 2f
$\boldsymbol{E_{A2}}$	4 mM	Threshold acetate concentration above which 1A01 dies	Supp. Fig. 4b, 4c
$\boldsymbol{E_{B1}}$	2.5 mM	Threshold acetate concentration for 3B05 to be unable to use acetate and acid-induced metabolites independently	Fig. 2f
$\boldsymbol{E_{B2}}$	3.5 mM	Soft threshold acetate concentration for 3B05 growth, even with both acetate and acid-induced metabolites	Model Parameter
$\boldsymbol{\Delta E}$	0.2 mM	Slope of the regulatory function for switch of 3B05 around E_{B2} from growth to lack of growth	Model Parameter
$\boldsymbol{K_{A,G}}$	10 µM	Monod constant for growth of 1A01 on GlcNAc	See Supp. Note 1
$\boldsymbol{K_{B,E}}$	10 µM	Monod constant for growth of 3B05 on Acetate	See Supp. Note 1
$\boldsymbol{K_{B,M}}$	10 µM	Monod constant for growth of 3B05 on acid-induced metabolites	See Supp. Note 1
$\boldsymbol{Y_{B,E}^{-1}}$	32 mM/OD	Amount of acetate consumed by 1 OD of growth by 3B05	Supp. Table 1
$\boldsymbol{Y_{A,E}^{-1}}$	9.4 mM/OD	Amount of acetate secreted during 1 OD of growth by 1A01	Supp. Table 1
$\boldsymbol{Y_{A,G}^{-1}}$	6 mM/OD	Amount of GlcNAc consumed by 1 OD of growth by 1A01	Supp. Table 1
$\boldsymbol{Y_{B,M}^{-1}}$	21 mM/OD	Amount of acid-induced metabolites consumed by 1 OD of growth by 3B05	Supp. Table 1 & stoichiometric constraints
$\boldsymbol{b}$	0.75	Fraction of carbon flux that 3B05 derives for its biomass from acetate	Fig. 5a, b and Supp. Table 1

Supplementary Table 7. Parameters used for the simulation of cross-feeding between *V. splendidus* sp. 1A01 and *N. phycotrophica* sp. 3B05; refer to **Supplementary Note 3** for details.

Supplementary Figures

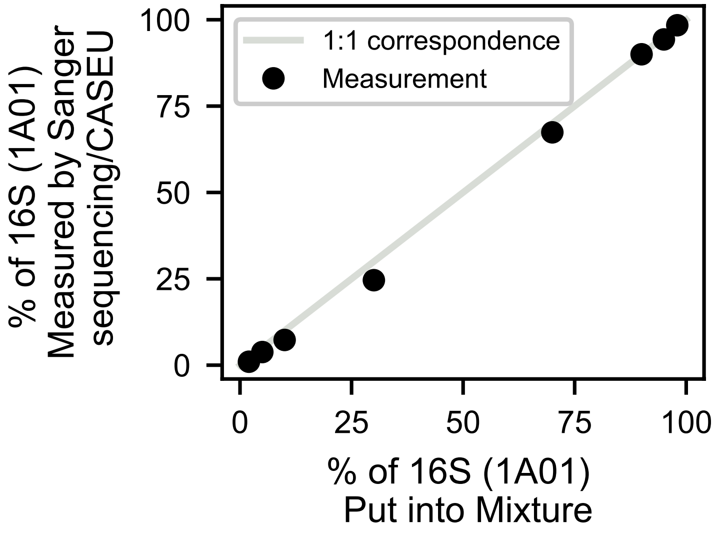

Supplementary Figure 1. Genotyping by 16S sequencing. Accuracy of Sanger sequencing electropherograms in determining the composition of 1A01 and 3B05 16S amplicon. Purified 16S amplicon of 1A01 and 3B05 were mixed in defined proportions using their concentrations measured by Nanodrop (expected concentration, light grey line). The mixtures were submitted for Sanger sequencing and the resulting electropherograms were deconvolved using CASEU[1] to determine the fraction of each type of 16S sequence. Source data are provided in the Source Data file.

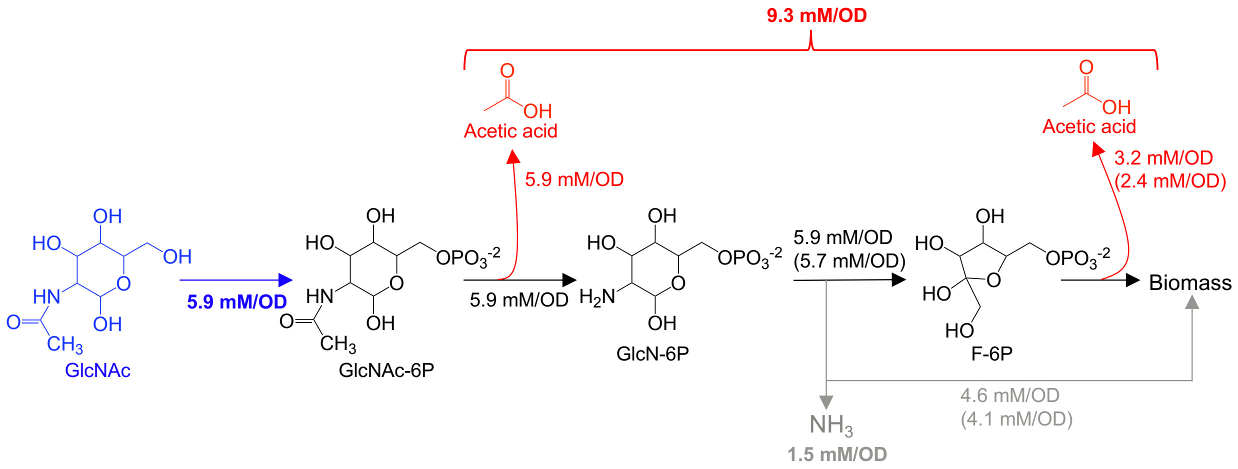

Supplementary Figure 2. Fluxes and yields for the growth of V. sp. 1A01 on GlcNAc. GlcNAc degradation pathway delineated previously for a *Vibrio* species[2] and for *E. coli*[3] depicting the excretion of acetic acid (red) and ammonium (grey). The yield numbers in bold are based on the measurements during exponential growth (**Fig. 1e**). The numbers in regular text are deduced based on stoichiometry and the measured values. The numbers in parenthesis are numbers obtained from similar measurements during exponential growth of 1A01 on glucose and ammonium (where 5.7 mM of glucose was consumed for 1 OD of cells). As shown in **Fig. 1e**, we measured 9.3 mM/OD of acetate excreted during growth on GlcNAc. Based on stoichiometry, we expect 6.1 mM/OD of acetate from the conversion of GlcNAc-6-P to GlcN-6-P (red arrow on the left) and 3.2 mM/OD from acetate overflow[4] (red arrow on the right). The latter is in line with what we measured for acetate excretion on glucose (2.4 mM/OD). Similarly based on stoichiometry, we expect 6.1 mM/OD of ammonium to be released from GlcN-P. We measured 1.5 mM/OD of ammonium excreted, implying 4.6 mM/OD of ammonium was assimilated into biomass, in line with the degree of ammonium assimilation we measured during growth on glucose (4.1 mM/OD). This analysis shows that growth yield on GlcNAc is similar to the yield on glucose and ammonium, so that the additional excretion of acetate and ammonium for the growth on GlcNAc compared to growth on glucose can be understood approximately as a result of the "extra" acetyl and amine groups accompanying the glucose in GlcNAc. In support of this interpretation, we found similar yield of acetate and ammonium due to excretion by *E. coli* growing on GlcNAc MOPS minimal medium with 0.4 M NaCl (**Supplementary Table 1**). The excretion yield for glucose is also provided there for reference.

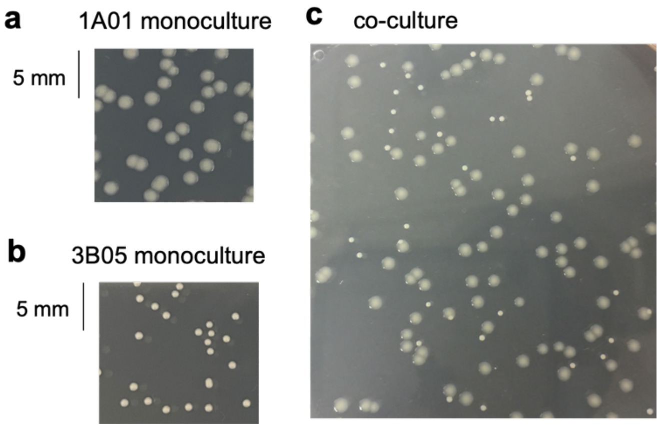

Supplementary Figure 3. Viability counts for 1A01 and 3B05. Colonies of 1A01 and 3B05 exhibit different morphologies. 1A01 monoculture **(a)**, 3B05 monoculture **(b)**, and 1A01/3B05 **(c)** co-culture growing in HEPES buffered medium was plated on 1.5% agar with rich medium (marine broth). After incubation of the plate for ~36 hr at 27°C, 1A01 and 3B05 colonies were visibly different. 1A01 colonies have a much larger area and an off-white color, while 3B05 colonies are punctate and white. These visual differences allow the estimation of viable cells of each type per OD·mL of co-culture when plating the co-culture.

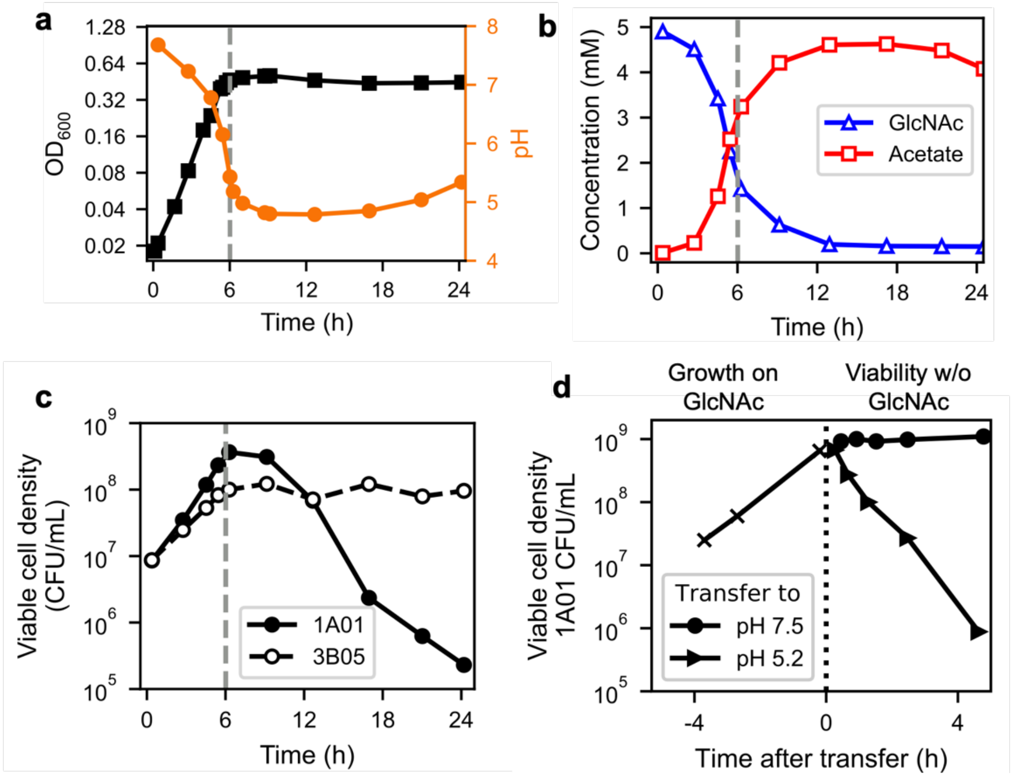

Supplementary Figure 4. Growth and death in the 1A01-3B05 co-culture during the first 24-h in weak buffer. The 1A01-3B05 co-culture was grown in 5 mM GlcNAc with the weak 2 mM bicarbonate buffer, inoculated at 1:1 ratio with an initial OD_{600} of 0.02. Various characteristics of the co-culture were monitored over the course of 24-hours after inoculation. **(a)** OD (black squares) and pH (orange circles) of the co-culture. **(b)** The concentrations of GlcNAc and acetate in the medium at various time points. **(c)** Viable cell counts for 1A01 (filled black circles) and 3B05 (open black circles). Data for the first 10-hours in panels (a)-(c) here are the same as those shown in main text **Fig. 2c-e**. The vertical dashed line in each panel indicates the time when growth is arrested (i.e., OD reaches saturation) in panel (a). Notice that the count of viable 1A01 cells dropped steeply several hours after growth arrest while the count of viable 3B05 remained approximately constant. **(d)** The death of 1A01 is characterized more closely in monoculture before and after exponentially growing 1A01 monoculture is transferred to medium with different pH: Before the transfer, 1A01 grew in HEPES-buffered medium with GlcNAc as the sole carbon and nitrogen source. Viable cell count (black x's) showed exponential growth consistent with growth rate obtained from OD measurement (**Fig. 1d**). At time t=0 (black dotted line), these cells were washed and resuspended into GlcNAc-free medium with 2mM bicarbonate buffer, with the pH set to 7.5 (black circles) or pH 5.2 (black triangles). In the latter case, 3.6 mM of acetic acid was added to lower the pH. Rapid drop of viable 1A01 cells was observed for the ones transferred to the medium at low pH but not at normal pH. Thus, low pH with acetate and lack of GlcNAc were sufficient for the rapid death of 1A01. Looking back at the data in panel (c), we see that the viability of 1A01 in the co-culture also started dropping after the depletion of GlcNAc (around 12h after inoculation, panel b). Source data are provided as in the Source Data file.

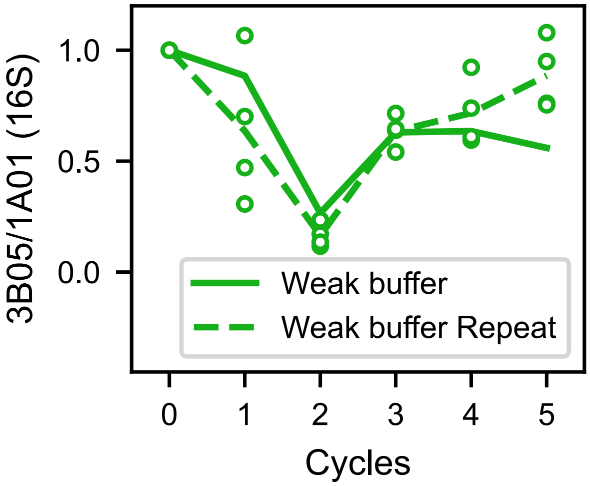

Supplementary Figure 5. The occurrence of substantial death of 1A01 while under acid stress (**Supp. Fig. 4**) raised the question of whether the coexistence seen in 24-h growth-dilution experiment under weak buffer (**Fig. 3a-c**) might have resulted from mutation and selection. We thus repeated the growth-dilution experiment using clones isolated from the end of 5 growth-dilution cycles. The replicated data are shown as green open circles, with the dashed green line connecting the mean of the data after each cycle. As the result is similar to that obtained using freshly streaked colonies (solid green line, reproduction of the weak buffer data shown in **Fig. 3a**), we conclude that evolution is not a concern here. Source data are provided in the Source Data file.

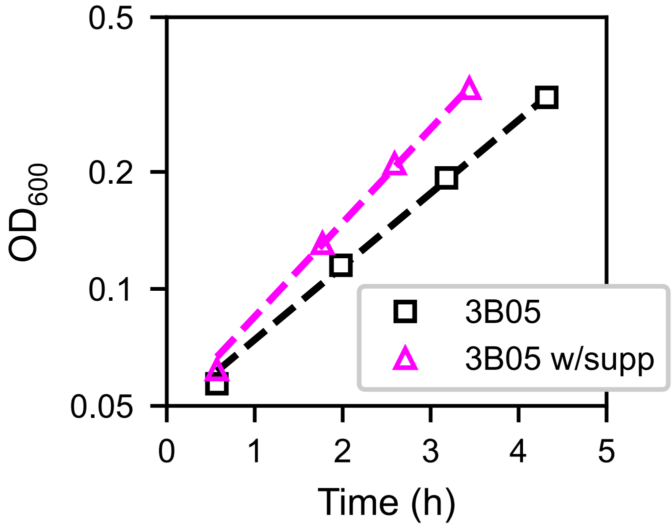

Supplementary Figure 6. Effect of metabolic supplement on the growth of 3B05. The magenta triangles indicate the growth of 3B05 monoculture in minimal medium with 0.35M NaCl and 40mM HEPES buffer, with 20 mM sodium acetate supplemented with 20 mM each of lactate, pyruvate, and glutamate, and with 10 mM NH_4Cl. The dashed magenta line through the triangles indicates an exponential fit to the growth curve. The black squares and dashed lines indicate growth of 3B05 in the same medium but without the supplement of lactate, pyruvate and glutamate. It is replicated from **Fig. 1f** for the ease of comparison. The best-fit parameters along with the standard deviation of the fits are summarized in **Supplementary Table 1**. Source data are provided in the Source Data file.

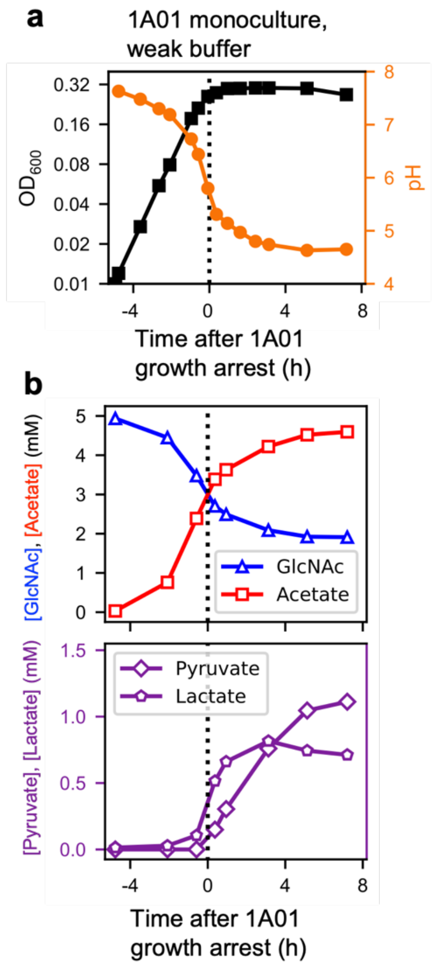

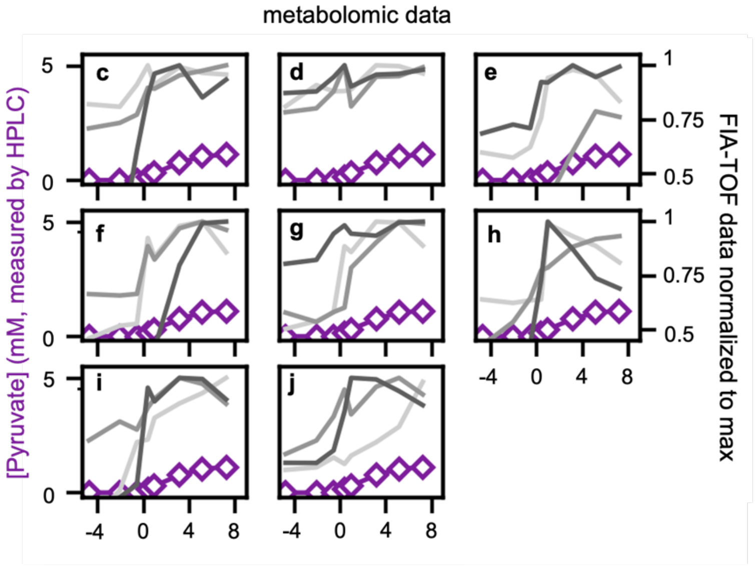

Legend

Panel	(light grey)	(grey)	(dark grey)
c	2-deoxyribose	2-hydroyglutarate	glycerate
d	2-aceto-lactate	lactate	2-ketoglutarate
e	1-amino-propan-2-one-3-phosphate	3-dihydroshikamate	3,4-hydroxy-phenylglycol
f	4-amino-butyrate	coumaraldehyde	4-hydroxybenzoate
g	5-oxoproline	Cis-aconitate	2-methylmaleate
h	Ribulose-5p	glycine	ascorbate
i	Aspartate-semialdehyde	Glutamate-semi	glutamate
j	GlcNAc-6P	Acetyl-glutamate	pyruvate

Supplementary Figure 7. Excretion of metabolites in 1A01 monoculture during growth on GlcNAc in weak buffer. 1A01 was grown in 5 mM GlcNAc in the weak 2 mM bicarbonate buffered medium. **(a)** shows OD (black squares) and pH (orange circles). **(b)** shows the GlcNAc and acetate concentrations (top panel), The black dotted line indicates the time at which 1A01 stops growing. Panels **(c)-(j)** show the results of FIA-QTOF-MS analysis of the same spent media samples analyzed in panel (b). The right y-axis indicates the measured intensity of each metabolic feature normalized by the max intensity of that feature during the time course of measurement. For the ease of visualization, each panel shows the result of three metabolic features, as grey, dark grey, and black lines. The corresponding metabolic feature for each line is given in the legend below. For reference, we also plotted the absolute concentration of pyruvate (purple diamonds) independently measured on the same samples using HPLC (left y-axis). Source data are provided in the Source Data file.

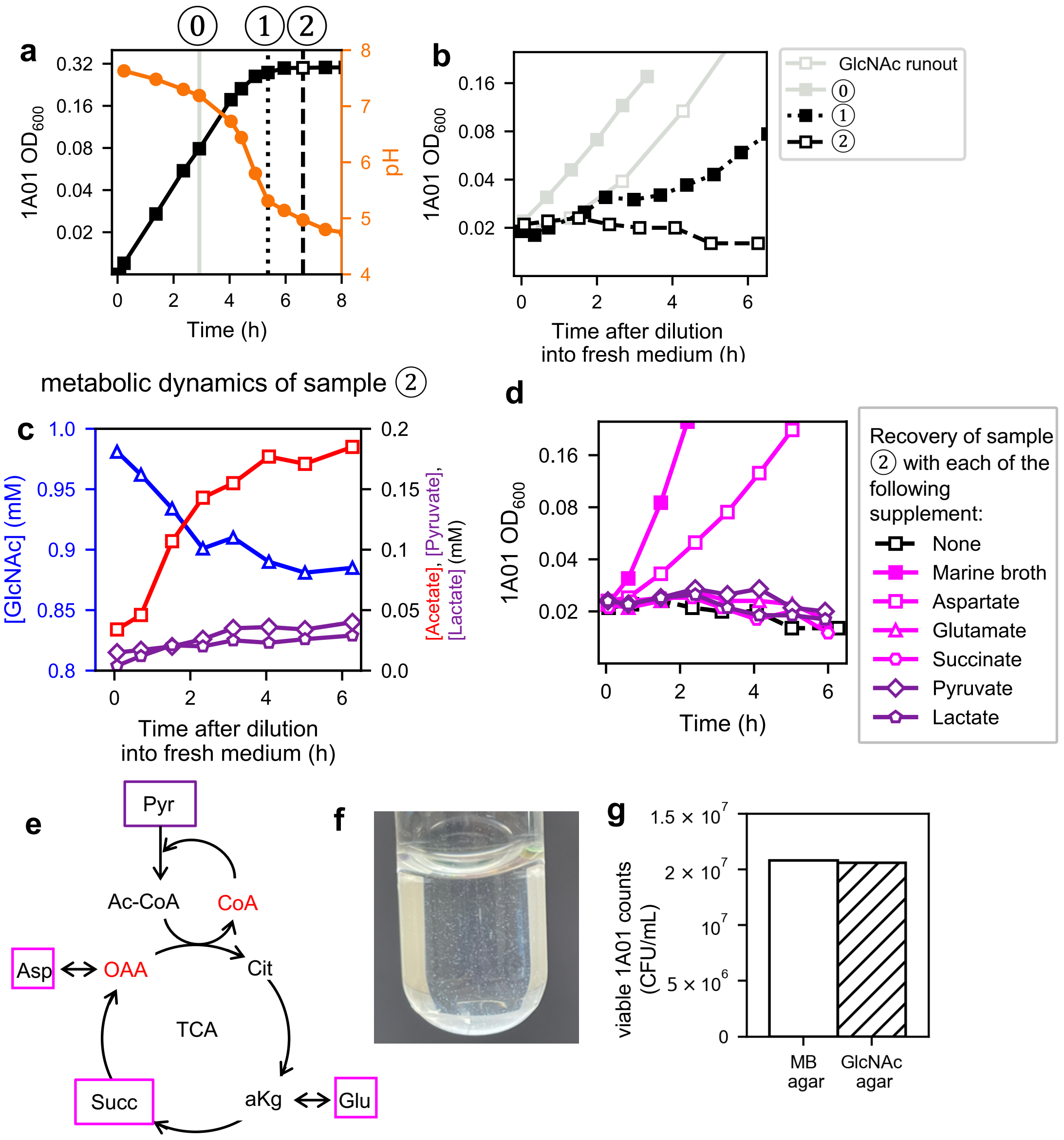

Supplementary Figure 8. Delay in 1A01 growth recovery after experiencing acetate stress. We hypothesize that the 6-hour lag by 1A01 after it was placed back into fresh medium at the start of the "stable cycle" (**Fig. 4a**) was due to the depletion of some of its excreted metabolites (taken up by 3B05) during the brief exposure to acetate stress in the previous cycle. To test this, we grew 1A01 monocultures in GlcNAc in weak buffer, and took the culture at various time during the self-acidification process to characterize the growth recovery dynamics upon resuspension into fresh GlcNAc medium at normal pH.

(a) We grew 1A01 monoculture (black squares) in 5 mM GlcNAc with the weak 2 mM bicarbonate buffer, and collected samples of culture at time <0.5 h and ~1.5 h after growth arrest (dotted and

dashed black lines, indicated by ① and ②, respectively), as well as during exponential growth (solid grey line, indicated by ⓪). Samples ① and ② attempt to capture transient exposure to low pH (orange symbols), while sample ⓪ is a control for normal pH. **(b)** The cells taken at different points in (a) were washed and resuspended in fresh GlcNAc medium with 2 mM bicarbonate, with pH ~7.5. Growth recoveries are shown for each case as indicated by the legend. We indeed observed a lag, whose duration increased with increased exposure to acid stress (duration and the value of pH) prior to resuspension. Open grey squares indicate another control of 1A01 cells that spent ~12 h in strongly buffered medium following GlcNAc runout without pH dropping. **(c)** For cells taken at ② in (a), although the cell density did not recover during the first 6 hours (open black squares in panel b), the concentrations of GlcNAc in the medium (blue triangles, left axis) was being depleted over time, while acetate, pyruvate, and lactate (red squares, purple diamonds, purple pentagons, respectively, right axis) accumulated in the medium. Note that the sum of the carbon content of the excreted metabolites, about 0.8 mM C, nearly matched that contained in the 0.1 mM of GlcNAc consumed during the period. The excreted metabolites were likely what fueled the growth of 3B05 in the co-culture during the first 6-h of the stable cycle where 1A01 did not grow (**Fig. 4a**). **(d)** The lag by cells taken at ② was relieved if GlcNAc was supplemented by fresh marine broth (filled magenta squares), indicating that acetate-stressed 1A01 cells were not intrinsically limited from re-growth and suggested the lack of certain key metabolites. The dominance of acetate in the excreted metabolites indicates a bottleneck for Ac-coA to enter the TCA cycle, possibly due to a low pool of oxaloacetate (oaa) which catalyzes the entry to TCA; see illustration in panel **(e)**. We tested this hypothesis by supplementing the resuspended cells with compounds closely related to TCA (outlined by magenta squares in panel **(e)**): Supplementation of 5 mM Aspartate, which is linked to oaa by a single reversible transamination reaction, effectively shortened the lag (open magenta squares). In contrast, 5 mM glutamate or 5 mM succinate (triangles and circles, respectively), as well as 5 mM pyruvate or 5 mM lactate (purple markers) did not show any effect in agreement with our hypothesis.

(f)-(g) We finally describe a different aspect of the lag phase, a moderate drop of 1A01 colony count when plating the culture sampled during the first 6 hours after dilution in the stable cycle (**Fig. 4a**). This drop is due to cell aggregation, not loss of cell viability: **(f)** Picture of co-culture taken during the lag phase of the stable cycle of the 24-h growth-dilution experiment in weak buffer, three hours after diluting into fresh GlcNAc medium: Macroscopic aggregates are seen in the culture. Thus, aggregation of 1A01 cells may account for all or a part of the drop in 1A01 colony count observed during the lag phase. **(g)** To show that viability is not lost during the lag phase, 1A01 cells were plated onto GlcNAc minimal medium plate and marine broth plate immediately following dilution of the stable cycle. On either plate, individual cells have no chance to aggregate. If exposure to GlcNAc minimal medium was somehow toxic to 1A01, we expect to see a drop in colony count on the GlcNAc minimal medium plate compared to marine broth plate, since the latter culture grew rapidly in marine broth without lag (filled magenta squares, **(d)**). However, we saw no difference in colony count between the two plate types. Thus, there is no death of 1A01 upon dilution into GlcNAc minimal medium.

Source data are provided in the Source Data file.

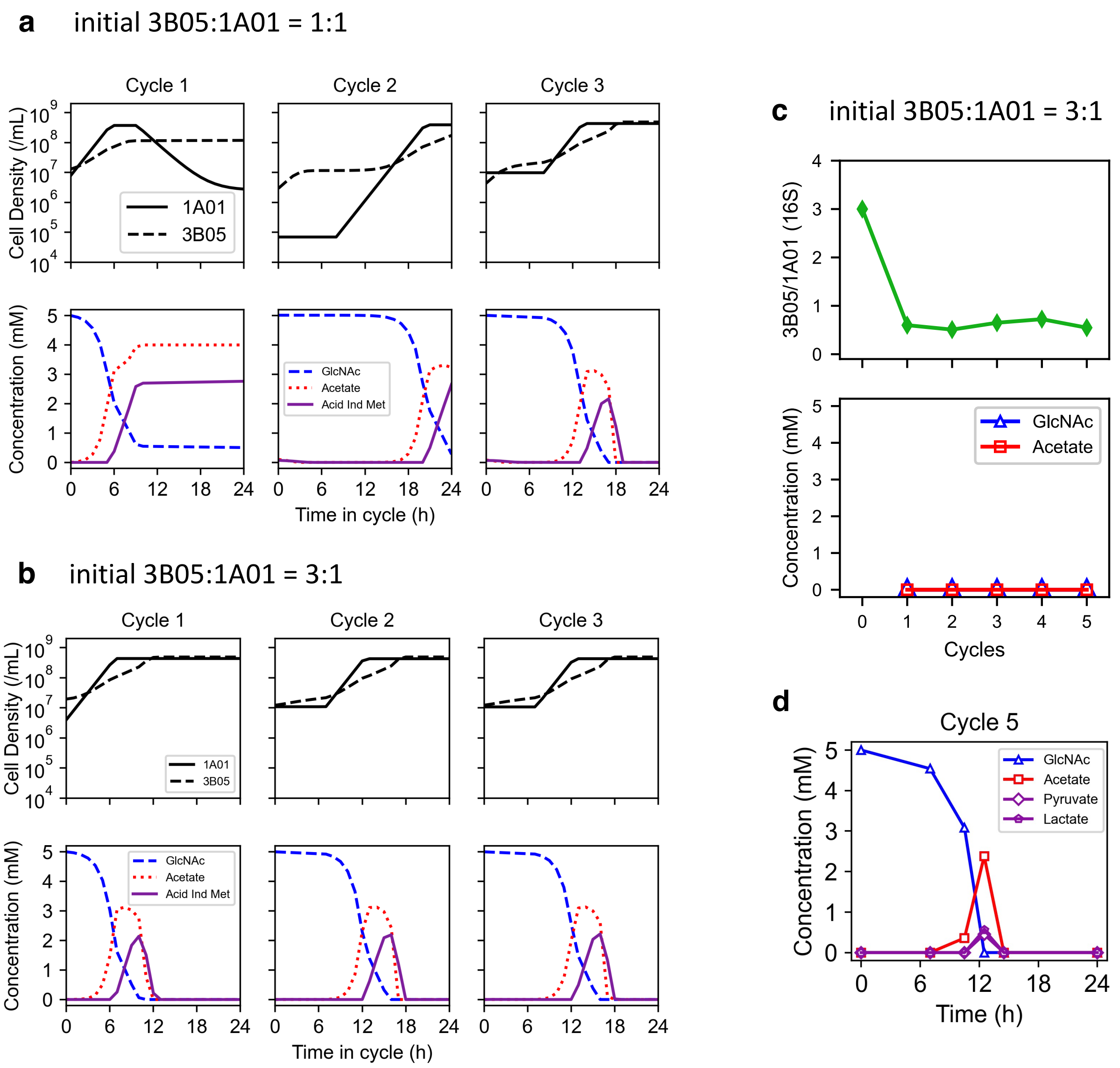

Supplementary Figure 9: Dynamics of approach to the stable cycle. Using the model of 1A01-3B05 cross-feeding dynamics described in **Supplementary Note 3**, we simulated the co-culture in 24h growth-dilution cycle starting with **(a)** 1:1 ratio and **(b)** 3:1 ratio of 3B05 to 1A01. The model predicts that the stable cycle is attained after two 24-h cycles for 1:1 starting ratio and after a single cycle for 3:1 starting ratio. The dynamics of the metabolites (GlcNAc, dashed line, acetate, dotted line, internal metabolites, solid line) during the stable cycle is predicted to be the same for different initial ratios. See **Supplementary Note 3** for a detailed description of the approach to the stable cycle. The predictions for the 1:1 initial ratio captured the experimental findings shown in main text **Fig. 3b, 3d, 4b**. The predictions for 3:1 initial ratio were tested experimentally in panels **(c)** and (d), with all other experimental conditions being identical to those described in the main text, i.e., for the 1A01-3B05 co-culture growing in minimal medium with 5 mM GlcNAc as the sole carbon and nitrogen source, buffered by 2 mM sodium bicarbonate, with 24-hour growth-dilution cycles at 40x dilution, and a total initial OD_{600} of 0.02. **(c)** Ratio of 16S reads (3B05:1A01), and the concentrations of GlcNAc (blue triangles) and acetate (red squares) were

measured at the end of each growth cycle. The data validated the predicted approach to the stable cycle after a single cycle. **(d)** The concentrations of GlcNAc, acetate, pyruvate (purple diamonds), and lactate (purple pentagons) on day 5 of a growth-dilution experiment that started with a 3:1 ratio of 3B05 to 1A01. The same acetate peak appeared transiently, at around t=12 h where GlcNAc was depleted, as seen in the stable cycle starting from 1:1 initial ratio (**Fig. 4b**). Thus, the same stable cycle is reached for both initial ratios of 3B05 to 1A01, despite very different transient dynamics. Source data are provided in the Source Data file.

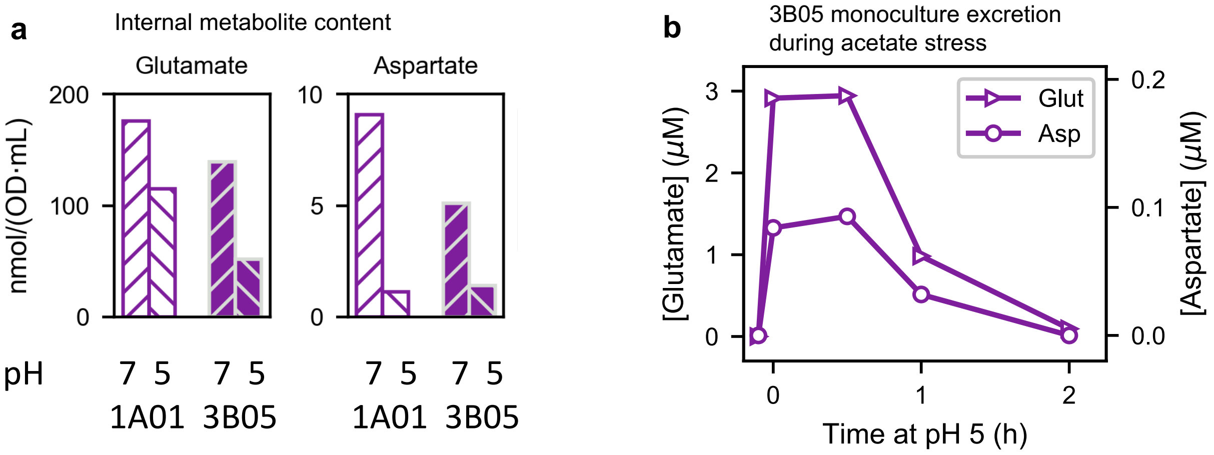

Supplementary Figure 10. The depletion of glutamate and aspartate for acid-stressed cells. **(a)** Measurement of internal content of glutamate and aspartate (see **Methods**) in 1A01 and 3B05 in facile conditions ('pH 7') and during acid stress with lower pH ('pH 5'). For 1A01, pH 7 cells were taken when the monoculture grown in 5 mM GlcNAc in the weak 2 mM bicarbonate buffered medium reached OD 0.1, and pH 5 cells were taken from the same culture when the pH dropped to 5 due to the buildup of acetate. (These correspond to sample ⓪ and ② , respectively, in **Supp. Fig. 8a**). For 3B05, we took a preculture growing exponentially in acetate in strong buffer, washed the cells, and resuspended into either 10 mM acetate in the weak 2 mM bicarbonate buffered medium (pH 7) or 4.5 mM acetate in the weakly buffered medium with the pH set to 5 (pH 5). The data were taken 1 h after resuspension. **(b)** Measurement of glutamate and aspartate in media before and after 3B05 was exposed to the acid stress described in **(a)**. Pyruvate and lactate concentrations were below detectable levels and are not shown. Source data are provided in the Source Data file.

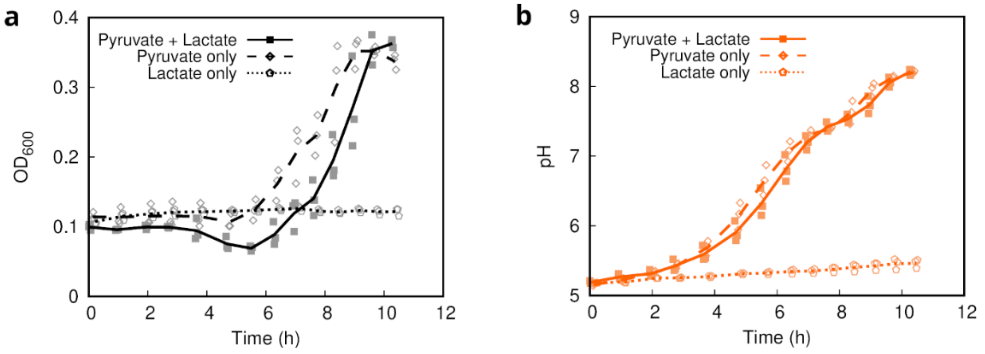

Supplementary Figure 11. Dependence of 3B05 deacidification on pyruvate and lactate. We grew *Neptunomonas phycotrophica* sp. 3B05 in weakly buffered minimal marine medium on acetate at pH ~5 supplemented with either 1 mM pyruvate and 1.5 mM lactate (Pyruvate + Lactate, closed squares), 2.5 mM pyruvate (Pyruvate only, open diamonds), or 2.5 mM lactate (Lactate only, open pentagons); see **Methods**. Points are data from individual replicates and the lines follow the mean values for each condition (Pyruvate + Lactate, solid; Pyruvate only, dashed; and Lactate only, dotted). The experiments here are similar[*] to those described in **Fig. 5a-c**, but with the supplementation of different combinations of pyruvate and lactate. **(a)** Growth curve (OD_{600} vs. time) for each condition. The data shows that growth supplemented by pyruvate-only is similar to that supplemented by both pyruvate and lactate, while supplementing lactate-only is insufficient for growth. In the case of supplement with both pyruvate and lactate, we believe both substrates are utilized by cells because the yield (maximum OD_{600} reached on 1.5 mM pyruvate and 1.0 mM lactate) is similar to that reached on 2.5 mM pyruvate alone. **(b)** Recovery of the pH of the culture for each condition. Each datum is linked (from the same culture) to the corresponding OD_{600} datum in the same condition taken at the same time in (a). Similar to the growth curve data, the pH recovery data shows that when supplemented only with pyruvate, the culture can deacidify the environment, much like when supplemented with both pyruvate and lactate. But lactate supplementation alone is insufficient for deacidification.

Lactate is a more reduced form of pyruvate. The inability to utilize lactate alone suggests difficulty in generating electron carriers such as NADH. This may be due to a reduced enzymatic activity or a surplus of reduction power in 3B05 cells under acetate stress. The latter is difficult to rationalize given the culture is growing in well-aerated conditions. However, as the main reaction converting lactate to pyruvate,

$$\text{lactate} + NAD^{+} \rightleftharpoons \text{pyruvate} + NADH + H^{+},$$

involves the generation of proton as a by-product, the reaction may be inhibited kinetically and thermodynamically at reduced pH. Indeed, the activity of the corresponding enzyme (Lactate Dehydrogenase) catalyzing this reaction in *E. coli* is reported to increase several-fold in the reverse direction at pH 6.4 compared to pH 7.5 [5]. This supports a pH-dependent inhibition of the conversion, which is relieved as growth recovers.

---

[*] The experiment with the supplement of both pyruvate and lactate (solid line) is close to a repeat of those in **Fig. 5a-c**. However, some quantitative differences are observed in the growth curves, e.g., a dip in OD_{600} before growth recovery. These differences could be due to slight changes in starting pH (pH 4.9 in **Fig. 5a** and pH 5.1-5.2 for the experiment here), or due to the absence of glutamate in the experiment here. Additionally, moderate degrees of cellular aggregation were observed in these experiments and are likely the cause of the observed decrease in OD_{600}.

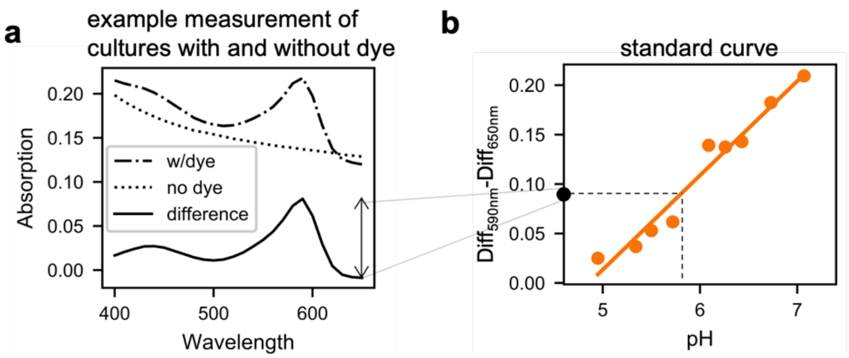

Supplementary Figure 12. Continuous pH measurement. To enable the continuous measurement of pH with a plate reader, we developed the following method. First, we initiated two cultures with identical inoculants. The only difference between the growth media in the two cultures was that one culture contained 0.0004% w/v bromocresol purple ('with dye') and the other did not ('without dye'). We measured the absorption spectrum of the two cultures every 15 min as the cells grew. **(a)** To calculate the pH at a particular time point, we first subtracted the spectrum of the culture 'with dye' (dash dotted line) from the spectrum of the culture 'w/o dye' (dotted line) to remove the scattering due to the cells and get the 'difference' (solid line). Next, we subtracted the difference value at 650 nm from that at 590 nm to calculate the height of the peak for the basic form of the dye. **(b)** This height was then converted to a pH using a standard curve measured using growth media adjusted to different, known pH values (slope = 0.10, y-intercept = -0.46, $R^2 = 0.96$). Source data are provided in the Source Data file.

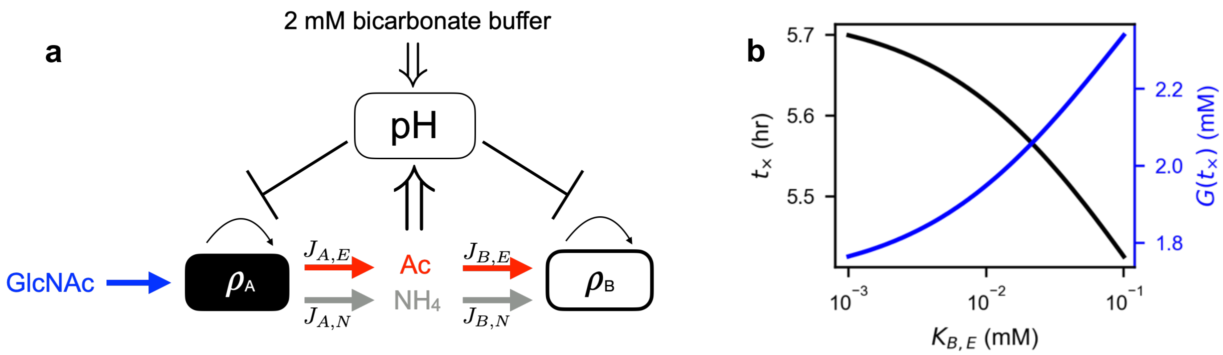

Supplementary Figure 13. Model of co-culture growth with stopping due to acetate buildup. **(a)** Schematic of model. The density of 1A01 (ρ_A) increases by taking up GlcNAc and turning it into biomass while excreting both acetate and ammonia with fluxes $J_{A,E}$ and $J_{A,N}$, respectively. The density of 3B05 (ρ_B) increases by taking up acetate and ammonia with fluxes $J_{B,E}$ and $J_{B,N}$ and turning it into biomass. The growth of both 1A01 and 3B05 are inhibited by acetate due to the weak buffering capacity of 2 mM bicarbonate. **(b)** Dependence of the co-culture stopping time ($t_\times$) and GlcNAc concentration at that time ($G(t_\times)$) on the Monod constant ($K_{B,E}$) for the growth of 3B05.

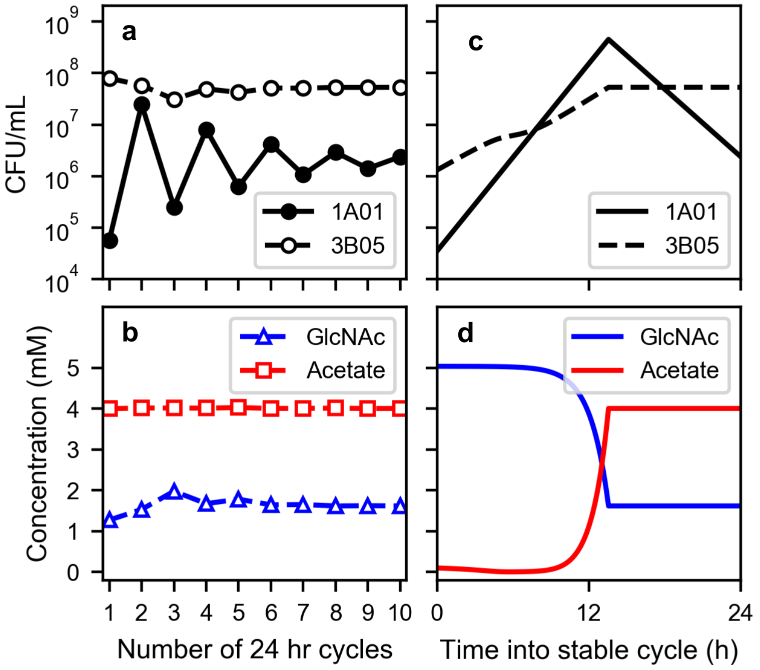

Supplementary Figure 14. Simulation results using the model of 1A01-3B05 cross-feeding including cell death as described above, applied to 24-h growth-dilution cycles with 5mM GlcNAc at the start of the cycle and 40x dilution. The density of live 1A01 cells is indicated by a solid black line and 3B05 by a dashed black line. The concentrations of acetate and GlcNAc are indicated by the red and blue lines, respectively. **(a)** and **(b)** show the simulation results at the end of each 24-h cycle. **(c)** and **(d)** show the simulation results during Cycle 10, after the co-culture has stabilized.

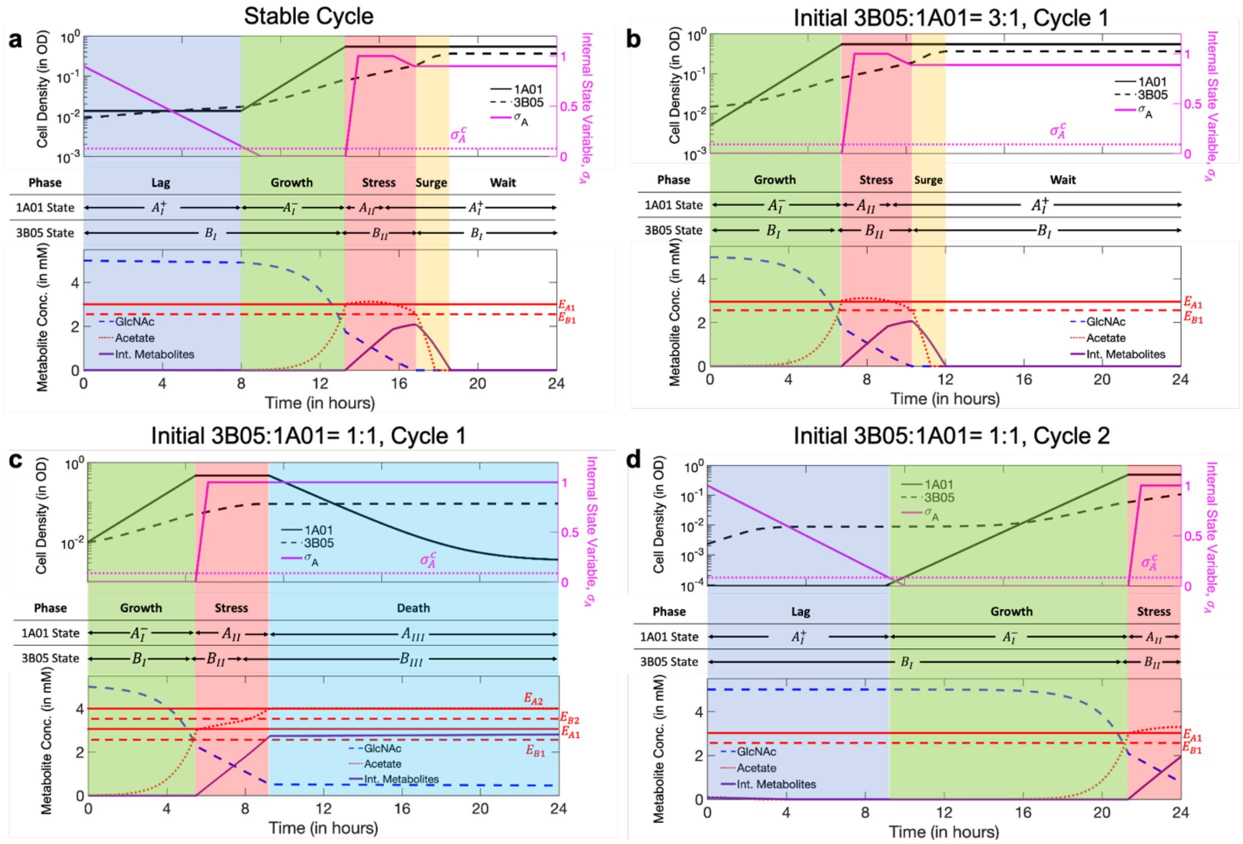

Supplementary Figure 15: Dynamics of cells and metabolites. In each panel, densities of 1A01 and 3B05 are shown on the top, and the concentrations of GlcNAc, acetate, and acid-induced metabolites (taken to be the sum of pyruvate and lactate) are shown in the bottom. Different colors indicate the different phases of the co-culture: lag (purple), growth (green), stress (pink), surge (yellow), wait (white). **(a)** the stable cycle; **(b)** first cycle with initial inoculant of 3B05:1A01 = 3:1; **(c), (d)** first two cycles with initial inoculant of 3B05:1A01 = 1:1.

Supplementary Notes

Supplementary Note 1: Effect of acetate built-up on the growth of co-culture

To determine whether the co-culture of 1A01 and 3B05 (abbreviated as A and B, respectively when needed) consumes all of the carbon in weakly buffered 2 mM bicarbonate medium with 5 mM GlcNAc, we constructed a kinetic model of growth and cross-feeding (**Fig. 13a**) parameterized using measurements on the single strains (**Supplementary Table 1**).

The growth rate of 1A01 depends on the GlcNAc concentration, $G(t)$, and the pH of the co-culture. The growth rate of 3B05 depends on the concentrations of acetate, $E(t)$, ammonium, $N(t)$, and the pH of the culture. As shown in **Fig. 2d, 2e**, both 1A01 and 3B05 stop growing at acetate concentrations above ~2.5-3 mM. Further, we note from **Fig. 2f**, that 3B05 is more sensitive to pH, and hence to increased acetate (since for our conditions, pH is a unique function of the acetate concentration, $E(t)$). For simplicity, we take the transition from growth to growth arrest to be a hard switch. Thus, for E below some threshold concentrations, $E_{B1} = 2.5$ mM and $E_{A1} = 3$ mM for 3B05 and 1A01 respectively, we take the growth rates to be of the Monod form, and no growth if $E(t)$ is above the thresholds. This leads to Eqs. (1.1) & (1.2) for the growth of 1A01 and 3B05, whose densities are denoted as ρ_A and ρ_B respectively,

$$\frac{d\rho_A}{dt} = \begin{cases} r_A \cdot f\left(G/K_{A,G}\right) \cdot \rho_A, & E < E_{A1} \\ 0, & E \geq E_{A1} \end{cases} \tag{1.1}$$

$$\frac{d\rho_B}{dt} = \begin{cases} r_B \cdot f\left(E/K_{B,E}\right) \cdot f\left(N/K_{B,N}\right) \cdot \rho_B, & E < E_{B1} \\ 0, & E \geq E_{B1} \end{cases} \tag{1.2}$$

with $f(x) \equiv x/(1 + x)$ describing the Michaelis dependence on the substrate concentration. For 3B05, we take growth to be simultaneously co-limited by the absence of either of the two substrates: acetate or ammonium. The multiplicative form for nutrient co-limitation can be thought of as a simple AND function requiring both nutrients to be present and is the predicted form for the limit of slow metabolic rates[9]. For the parameters of the Monod functions, the maximum growth rates r_i are taken from the batch culture measurements reported ($r_A = 0.7/\text{h}$ and $r_B = 0.35/\text{h}$ for weak buffer; see **Supplementary Table 1)**. The Michaelis constants $K_{A,G}$, $K_{B,E}$, and $K_{B,N}$ are very small ($\ll 1$ mM) given how abruptly the cultures cease growth when each nutrient runs out. For our calculations, they are taken to be 10 µM, but their precise values do not drastically affect the results as we will discuss below.

The dynamics of the metabolite concentrations are given by Eqs. (1.3)-(1.5). The uptake and excretion fluxes of metabolite m by species i, denoted as $J_{i,m}$, are given by $\lambda_i \rho_i / Y_{i,m}$, where $\lambda_i \equiv r_i \cdot f(x_i/K_{i,m})$ are the actual growth rates, and $Y_{i,m}$ (in unit of OD/mM) are the yield factors or the excretion factors, whose inverse values are listed in **Supplementary Table 1**.

$$\frac{dG}{dt} = -\frac{\lambda_A \rho_A}{Y_{A,G}} \equiv -J_{A,G}, \tag{1.3}$$

$$\frac{dE}{dt} = \frac{1}{Y_{A,E}} \frac{d\rho_A}{dt} - \frac{1}{Y_{B,E}} \frac{d\rho_B}{dt} = J_{A,E} - J_{B,E}, \quad (1.4)$$

$$\frac{dN}{dt} = \frac{1}{Y_{A,N}} \frac{d\rho_A}{dt} - \frac{1}{Y_{B,N}} \frac{d\rho_B}{dt} = J_{A,N} - J_{B,N}. \quad (1.5)$$

We simulated the 1A01-3B05 co-culture using Eqs. (1.1) -(1.5), starting with a 1:1 ratio at OD = 0.02 as was done in the experiment. As shown in **Fig. 2h**, the model predicts that after a short period, the acetate concentration increases exponentially and reaches the stopping concentration for 3B05 (E_{B1}) after ~6 h. Although 1A01 has a slightly larger stopping concentration (E_{A1}), once 3B05 stops growing and stops taking up acetate, acetate accumulates even more rapidly and 1A01 stops growing very shortly after. So, the two strains essentially stop growing at the same acetate concentration, $E \approx E_{B1}$. At this point, ~2 mM GlcNAc remains though the co-culture has stopped growing. During the 6-h growth period, the densities of 1A01 and 3B05 are expected to increase by 64-fold and 8-fold respectively, based on their maximum growth rates, such that the ratio of 3B05 to 1A01 cells has dropped from 1:1 at the start to 1:8 when growth ceases.

As mentioned above, the values of the Monod constants $K_{A,G}$, $K_{B,N}$, and $K_{A,E}$ are not known, other than that they are well below 1 mM. To assess the sensitivity of the co-culture dynamics shown in **Fig. 2g, 2h** to the values of these parameters, we note first that the dependence on $K_{A,G}$ and $K_{A,N}$ are completely negligible: $K_{A,G}$ is negligible because throughout the simulation the concentration of GlcNAc stayed above 1 mM which much exceeds $K_{A,G}$. $K_{B,N}$ is negligible compared to the ammonium concentrations as ammonium accumulates, indicating that it does not limit 3B05 growth in our experiments. However, $K_{B,E}$ does affect the outcome moderately. We varied $K_{B,E}$ from 1 to 100 μM while keeping $K_{A,G}$ and $K_{B,N}$ constant. The two key outputs of the model are plotted against $K_{B,E}$, the time at which the co-culture stops due to acetate build-up, $t_\times$, and the amount of GlcNAc remaining in the medium, $G(t_\times)$ (**Fig. 13b**). The plots show that $t_\times$ changed by less than 10% (black line), with the remaining GlcNAc concentration close to ~2 mM (blue line). Thus, the dynamics of the coculture are not significantly affected by the precise values of the Monod constants.

Supplementary Note 2. Model of growth-dilution cycles including cell death

In **Supplementary Fig. 4**, we showed that 1A01 cells died rapidly when exposed to low pH in the absence of GlcNAc. Such a death would decrease the average growth rate of 1A01 over a cycle and possibly allow 3B05 to recover and de-acidify the environment. Can the death of 1A01 explain the stable coexistence the 1A01-3B05 co-culture in weak buffer (**Fig. 3b**)? In this note, we examine this possibility based on the single strain characteristics measured in **Fig. 1** and **Fig. 2f**, together with the death characteristics in **Supp. Fig. 4d**. Our model for the co-culture is identical to that presented **in Supplementary Note 1** except that when the acetate concentration E reaches a threshold of $E_{A2} \approx 4$ mM, 1A01 starts dying with a death rate $\delta_A = 0.5/\mathrm{h}$ (**Supp. Fig. 4d**). This is implemented with the modification of Eq. (1.1) in **Supplementary Note 1** to the following:

$$\frac{d\rho_A}{dt} = \begin{cases} r_A \cdot f\left(G/K_{A,G}\right)\rho_A, & E < E_{A2} \\ -\delta_A \cdot \rho_A, & E \geq E_{A2}. \end{cases} \tag{2.1}$$

To simulate the growth-dilution cycles, the model now keeps track of the cycles. At the start of new cycle, all the metabolite concentrations and cell densities obtained at the end of period (24 hours) of the previous cycle were divided by the dilution factor (40-fold). In addition, 5 mM of GlcNAc was added upon dilution. The simulation results are shown in **Fig. 14a-d.** They show that the model with cell death is able to produce the co-existence of 1A01 and 3B05. The effect of cell death can be understood as follows: since the growth rate for 3B05 on acetate in the weakly-buffered medium is 0.35 h^{-1}, it needs at least 11.8 h to grow 40x to make up for the dilution at the end of a cycle. The rapid death of 1A01 means that, following dilution, it needs to grow ≫40x to maintain its population. Thus, 1A01 must undergo additional doublings until it reaches densities that can acidify the culture. This delays acetate buildup and the ensuing stoppage of coculture growth, thereby giving 3B05 more time to replicate and catch up for the 40x density increase required for coexistence. Quantitatively, our model shows that coexistence can be achieved in a stable cycle with growth occurring for the first ~13 h (panels c and d), as opposed to the first cycle where growth was limited to the first 6 hours (**Supp. Fig. 4c**). This results in a reduced period of growth arrest and hence a reduced drop in the viable 1A01 cells, such that in one cycle 1A01 and 3B05 each has just enough time to increase its density 40x. Thus, the death of a faster species can effectively delay the onset of toxicity, thereby enabling a slower cross-feeder to avoid extinction.

In this model, the key to the co-culture finding a stable cycle is that it stops growing prematurely, i.e., before the full consumption of GlcNAc. This point can be adjusted dynamically until a stable cycle is reached where the growth period for 1A01 is just enough to compensate for its death and the dilution factor. However, our data on GlcNAc and acetate at the end of each cycle of the 24-h growth-dilution experiment in weak buffer (**Fig. 3d**) showed major differences from the simulation result here: More acetate and less GlcNAc than predicted were found at the end of Cycle 1, and, once the co-culture stabilized, GlcNAc and acetate were entirely consumed whereas significant amount remained in the simulation (**Fig. 14b**). Also, the ratio of viable counts of 1A01 is comparable to 3B05 (**Fig. 3c**) whereas it is more than 10x lower than 3B05 in this model (**Fig. 14a**). Therefore, death of 1A01 modeled in this simulation cannot be the cause of stable coexistence in weak buffer that was observed experimentally.

Supplementary Note 3. Dynamical Model of acid-induced cross-feeding

The results of the simulations of the models in **Supplementary Note 1, 2** demonstrate that growth arrest, and even death, are not sufficient to account for the coexistence of 1A01 and 3B05, nor the complete utilization of the carbon source. The other experimental observation that allows 3B05 to recover after acidification of the environment is acid-induced metabolic cross-feeding between 1A01 and 3B05. Is acid-induced metabolic cross-feeding as has been characterized at the single-strain level in the main text (**Fig. 5**), together with the growth characteristic established in **Fig. 1, Supp. Fig. 4** and used in **Supplementary Note 1, 2**, sufficient to explain the coexistence of 1A01 and 3B05 and the complete utilization of the carbon source in the stable cycle (**Fig. 3b, 3d**)? Can we also account for the dynamical observations, e.g., the crash of the co-culture during the first 24 hours (**Supp. Fig. 4c**), the approach to the stable cycle shown in **Fig. 3b, 3d**, and the stable cycle dynamics itself (**Fig. 4a, 4b**)? We explore these possibilities by constructing in this Note a dynamical model of acid-induced metabolic cross-feeding between 1A01 and 3B05 based on the experimentally observed phenomena and parameters reported in the main text.

In addition to the densities of 1A01 and 3B05 cells, $\rho_A(t)$ and $\rho_B(t)$, respectively, and the metabolite concentrations, GlcNAc $G(t)$ and acetate $E(t)$, as discussed above, we also track the concentration of the sum of the major acid-induced metabolites in the culture (pyruvate and lactate), denoted by $M(t)$, which are cross fed during stress (**Fig. 4e**). We do not include the ammonium concentration here for simplicity as it is not the limiting nutrient in the coculture growth of 3B05.

Our model is set up in the general form of a consumer-resource model: 1A01 takes up GlcNAc with rate $\mu_{A,G}$, excretes acetate and acid-induced metabolites with rate $\mu_{A,E}$, $\mu_{A,M}$ respectively, and grows (or dies) at rate λ_A; 3B05 takes up acetate and acid-induced metabolites at rates $\mu_{B,E}$ and $\mu_{B,M}$ respectively and grows with rate λ_B. The population dynamics are therefore described straightforwardly as:

$$\frac{d\rho_A}{dt} = \lambda_A(G; E, \sigma_A) \cdot \rho_A, \tag{3.1}$$

$$\frac{d\rho_B}{dt} = \lambda_B(E, M; E) \cdot \rho_B, \tag{3.2}$$

$$\frac{dG}{dt} = -\mu_{A,G}(G; E, \sigma_A) \cdot \rho_A, \tag{3.3}$$

$$\frac{dE}{dt} = \mu_{A,E}(G; E, \sigma_A) \cdot \rho_A - \mu_{B,E}(E, M; E) \cdot \rho_B, \tag{3.4}$$

$$\frac{dM}{dt} = \mu_{A,M}(G; E, \sigma_A) \cdot \rho_A - \mu_{B,M}(E, M; E) \cdot \rho_B. \tag{3.5}$$

However, the rate functions, i.e., λ_i for species $i \in \{A, B\}$, and $\mu_{i,m}$ for the uptake or excretion of metabolite $m \in \{G, E, M\}$, cannot be taken to be independent of the environment, as the physiological states of the two species (which determine the growth characteristics and

environmental interactions) depend on the environment. Thus, the forms of the rate functions need to account for such dependences. In Eqs. (3.1) – (3.5), we indicated the two types of dependences of the rates, λ_i and $\mu_{i,m}$, separated by a semicolon: The value of each rate for species i depends on the concentration of the metabolites it takes up: G for 1A01 and M, E for 3B05; these are the entries that appear before the semicolon. The entries after the semicolon indicate factors that affect the physiologies of the species. The latter factors include not only E, which reflects the degree of acetate stress, but also another variable σ_A, which is an internal variable reflecting the depletion of other acid-induced metabolites and which affects the growth recovery of 1A01.

The forms of the rate functions λ_i and $\mu_{i.m}$ will be described in detail below, together with the dynamics of σ_A and the values of the parameters used. To simulate the growth-dilution cycles, Eqs. (3.1) – (3.5) are integrated numerically using the Finite Difference method and a step size of 0.36 seconds, starting with the initial conditions for the metabolites ($G(0) = 5$ mM, $E(0) = 0, M(0) = 0$) and the specified initial ratio of the two species (with $\rho_A(0) + \rho_B(0) = 0.02$ OD). After integrating these equations for 24 h, all the metabolite concentrations and the species densities are divided by the dilution factor (40-fold) and 5 mM is added to the GlcNAc concentration. The integration for the 24 h and the dilution at the end of the cycle is then repeated for five cycles. The simulation results for the viable cell density (cell/mL) reported in **Fig. 6** and **Supp. Fig. 9** were calculated by multiplying the OD from the simulation with the conversion factors (CFU/OD/mL) in **Supplementary Table 1**.

Forms of the rate functions for 3B05. In our experiments, 3B05 exhibited three distinct forms of growth depending on the environmental acidity and nutrient supplement as we describe below.

1. Under normal pH, 3B05 can grow on either acetate alone or on acetate, pyruvate, and lactate, with faster growth when pyruvate and lactate are present (**Supp. Fig. 6**). We describe these dependences by a simple sum of Monod growth functions, i.e.,

$$\lambda_B = r_{B,E} f\left(E/K_{B,E}\right) + r_{B,M} f\left(M/K_{B,M}\right), \quad \text{at normal pH} \tag{3.6a}$$

where $r_{B,E}$ and $r_{B,M}$ are the maximum growth rate of 3B05 on acetate and on the acid-induced metabolites alone respectively, $f(x) \equiv x/(1+x)$ describes the Michaelis dependence on the substrate concentration, and $K_{B,E}$, $K_{B,M}$ are the respective Monod constants. The value of $r_{B,E}$ is taken from measurements (**Fig. 1f**; see also **Supp. Table 1**) to be 0.35/h. $r_{B,M}$ is taken to be 0.2/h, such that the high growth rate of 0.55/h observed for growth on acetate, pyruvate, and lactate (**Supp. Fig. 6**) can be explained by additive growth on both substrates.

As discussed in **Supplementary Note 1**, the Monod constants are very small given how abruptly the cultures cease growth when each nutrient runs out. For our calculations, they are taken to be 10 μM but the results of this model are again found to be insensitive to the values of the Monod constants as the key metabolic processes occur when the concentrations of the metabolites are well above the experimentally determined upper limits of these constants. In this regime, the nutrient uptake rates by 3B05 are simply

$$\mu_{B,E} = r_{B,E} f\left(E/K_{B,E}\right)/Y_{B,E}, \tag{3.6b}$$

$$\mu_{B,M} = r_{B,M} f\left(M/K_{B,M}\right)/Y_{B,M}, \tag{3.6c}$$

with the mass conservation condition $\mu_{B,E} \cdot Y_{B,E} + \mu_{B,M} \cdot Y_{B,M} = \lambda_B$. The value of $Y_{B,E}$ was determined experimentally (**Supplementary Table 1**). We take $Y_{B,M} = \frac{3}{2} Y_{B,E}$ as each molecule of acetate has 2 carbon atoms, while each molecule of pyruvate and lactate has 3 carbon atoms.

2. At low pH, 3B05 does not grow even when acetate or other metabolites are abundant; see e.g., **Fig. 5a**. Thus, we take

$$\lambda_B = \mu_{B,E} = \mu_{B,M} = 0 \quad \text{at low pH.} \tag{3.7}$$

3. Crucially for deacidification, there exists an intermediate regime where 3B05 can grow on acetate, but only with the supplement of pyruvate and lactate (**Fig. 5a-c**). We model this simultaneous colimitation on acetate and the cross-fed metabolites by a product of Monod functions, i.e.,

$$\lambda_B = r_B^+ f\left(E/K_{B,E}\right) \cdot f\left(M/K_{B,M}\right), \quad \text{at intermediate pH,} \tag{3.8a}$$

where r_B^+ is the growth rate in this phase. As with Eq. (1.2), the multiplicative form for nutrient co-limitation is a simple continuous AND function requiring both nutrients to be present and is the predicted form for the limit of slow metabolic rates[6]. The range of possible values of r_B^+ is discussed below. In this intermediate regime, the nutrient uptake rates take on the form

$$\mu_{B,E} = b\, r_B^+ f\left(E/K_{B,E}\right) f\left(M/K_{B,M}\right)/Y_{B,E}, \tag{3.8b}$$

$$\mu_{B,M} = (1-b)\, r_B^+ f\left(E/K_{B,E}\right) f\left(M/K_{B,M}\right)/Y_{B,M}, \tag{3.8c}$$

such that the mass conservation condition $\mu_{B,E} \cdot Y_{B,E} + \mu_{B,M} \cdot Y_{B,M} = \lambda_B$ still holds. The factor b reflects the fraction of carbon flux that 3B05 derives for its biomass from acetate. Plotting the OD and metabolite date shown in **Fig. 5a, b** and comparing to the carbon yield of 3B05 on acetate alone (**Supplementary Table 1**), we estimate $b \approx 0.75$.

A key challenge in completing the model is to specify quantitatively how the system transitions from one regime of pH to another. As this information is difficult to obtain experimentally (requiring the setting of acetate to different fixed levels while 3B05 consumes it), we used simplified forms for transitions as already described in **Supplementary Note 1**: The regime of normal pH applies for acetate concentration below a threshold value, E_{B1}. However, for $E > E_{B1}$, the transition between the intermediate and low pH regimes is much more complicated and possibly path-dependent, or even density-dependent. To recover the experimental phenomenological results, we implement this transition by a soft cutoff, $\theta(E_{B2} - E) \equiv \frac{1}{2}(1 + \tanh((E_{B2} - E)/\Delta E))$, around a second threshold, E_{B2}, such that for $E \gg E_{B2}$, the rates λ_B and $\mu_{B,m}$ all vanish. A hyperbolic tangent function was chosen for functional simplicity, and the parameter values for r_B^+, E_{B2}, and ΔE were taken to be round numbers in the range determined by experimental constraints. For example, the dynamic nature of the data in **Fig. 5a-c** does not allow extraction of a steady-state growth rate, but helps us to place a bound above ~0.1/h. We also see from the data in **Fig. 4c** that the growth of 3B05 slowed down from pre-stressed growth (0.35/h,

dot-dashed line) when approaching the acetate peak before picking up past the peak. Thus, we constrain the parameter r_B^+ to the range between 0.1/h and 0.35/h and we take $r_B^+ = 0.25/\mathrm{h}$. Similarly, in practice we found the transition width ΔE can be quite small and in our simulations, we use $\Delta E = 0.2$ mM, which is only 20% of the difference between the threshold values $E_{B2} - E_{B1} = 1$ mM. Altogether, the rate functions in the different regimes are summarized in **Supplementary Table 5**. We shall discuss the values of the thresholds E_{B1}, E_{B2} in conjunction with similar thresholds for 1A01 after describing the rate functions for 1A01 below.

Forms of the rate functions for 1A01. The growth of 1A01 is found to depend on both the acetate concentration (E) and the internal state of the cells, σ_A. Here we describe the forms of the rate functions λ_A and $\mu_{A,m}$ for 1A01 in our model, which we take to be in four possible states.

1. At normal pH, 1A01 is in exponential phase. Thus, it grows on GlcNAc with a Michaelis dependence on the GlcNAc concentration and $K_{A,G}$ being the Monod constant; the latter is again taken to be 10 μM, with the results of the model insensitive to the exact value used (see above and **Supplementary Note 1**). Thus,

$$\lambda_{A,G} = r_A f(G/K_{A,G}), \quad \text{at normal pH.} \tag{3.9}$$

In this regime, the nutrient uptake rate by 1A01 ($\mu_{A,G}$) is simply given by mass conservation ($\lambda_{A,G} = Y_{A,G}\mu_{A,G}$) and acetate is excreted by 1A01 as a by-product with excretion rate given by $\mu_{A,E} = \lambda_{A,G}/Y_{A,\mathrm{E}}$. No acid-induced metabolites are excreted in this state. We take $r_A = 0.7/\mathrm{h}$, $Y_{A,G}^{-1} = 6$ mM/OD, and $Y_{A,E}^{-1} = 9.4$ mM/OD based on data in **Supplementary Table 1**. Thus,

$$\mu_{A,G} = -r_A f(G/K_G)/Y_{A,\mathrm{G}}, \tag{3.10a}$$

$$\mu_{A,E} = r_A f(G/K_G)/Y_{A,\mathrm{E}}, \tag{3.10b}$$

$$\mu_{A,M} = 0. \tag{3.10c}$$

2. At very low pH, we find experimentally that 1A01 dies at a constant rate, and there is no accompanying substrate consumption or production. We take $\delta_A = 0.5/\mathrm{h}$ based on the data in **Supp. Fig. 4c, 4d**. Thus,

$$\lambda_{A,G} = -\delta_A, \text{ at very low pH} \tag{3.11a}$$

$$\mu_{A,G} = \mu_{A,E} = \mu_{A,M} = 0. \tag{3.11b}$$

3. At intermediate pH values, we find that there is no growth or death of 1A01 (**Fig. 5d**), i.e.,

$$\lambda_{A,G} = 0, \text{ at intermediate pH.} \tag{3.12}$$

However, in this pH range, 1A01 cells excrete acid-induced metabolites while consuming GlcNAc (and still producing acetate as a by-product); see **Fig. 5e,f**. We model this excretion process by the same Michaelis dependence on GlcNAc concentration:

$$\mu_{A,G} = \mu_{A,G}^{str} f(G/K_G), \quad (3.13a)$$
$$\mu_{A,E} = \mu_{A,\mathrm{E}}^{str} f(G/K_G), \quad (3.13b)$$
$$\mu_{A,M} = \mu_{A,\mathrm{M}}^{str} f(G/K_G). \quad (3.13c)$$

The values of $\mu_{A,G}^{str}$, $\mu_{A,E}^{str}$, and $\mu_{A,M}^{str}$ were determined from the **Fig. 5d-f**.

4. However, 1A01 cells do not always grow even in the normal pH regime (**Fig. 4a)**. For 1A01 cells that have experienced acetate shock, we find a substantial lag phase of at least 6 hours which likely results from the depletion of key metabolites not related to pyruvate and lactate (**Supp. Fig. 8**). To model the effect of the latter, we introduce one additional variable that describes the internal state (of metabolite depletion), σ_A, whose value is normalized to be between 0 and 1. We can think of high values of σ_A as corresponding the case of severe depletion of these additional metabolites, and low values of σ_A to correspond to the normal state. Thus, 1A01 cells that have recently experienced acetate stress and are in the process of recovering from acetate stress (in which case $\sigma_A > \sigma_A^c$) do not grow even if the pH is normal:

$$\lambda_{A,G} = 0, \text{ for } \sigma_A > \sigma_A^c. \quad (3.14)$$

In this state, 1A01 cells still excrete pyruvate and lactate while consuming GlcNAc (and still excretes acetate as a by-product); see **Supp. Fig. 8c**. We model the excretion process here again with a Michaelis dependence on GlcNAc concentration:

$$\mu_{A,G} = \mu_{A,G}^{lag} f(G/K_{A,G}), \quad (3.15a)$$
$$\mu_{A,E} = \mu_{A,\mathrm{E}}^{lag} f(G/K_{A,G}), \quad (3.15b)$$
$$\mu_{A,M} = \mu_{A,\mathrm{M}}^{lag} f(G/K_{A,G}). \quad (3.15c)$$

The values of $\mu_{A,G}^{lag}$, $\mu_{A,E}^{lag}$, and $\mu_{A,M}^{lag}$ were determined from **Supp. Fig. 8a-c** during the growth lag. It must be noted that our results hold even if we take $\mu_{A,m}^{lag} = \mu_{A,m}^{str}$ for each metabolite m, and we distinguish between the two values to maintain fidelity with the reported experimental results.

All the rate functions pertaining to 1A01 are summarized in **Supplementary Table 6**, with the transition between normal, intermediate, and low pH occurring at a threshold E_{A1} and E_{A2}.

Next, what determines the transitions between the different physiological states enumerated above? As discussed in **Supplementary Note 1**, both 1A01 and 3B05 stop growing (with 3B05 being more sensitive to acetate concentrations) above 2.5-3 mM, (based on data shown in **Supp. Fig. 4c, 4d**). Accordingly, $E_{A1}$was taken to be 3mM and E_{B2} was taken to be 2.5 mM. For E_{A2}, we note that as shown in **Supp. Fig. 4c**, 1A01 starts dying around 9-10 hours, which corresponds to around 4 mM of acetate as shown in **Supp. Fig. 4b**. Thus, we take E_{A2} to be 4 mM. As discussed above, the cessation of growth of 3B05 in the presence of pyruvate and lactate is much more complicated, and we only attempt to recover the phenomenological features in our model with a constant rate and a simple tanh-like switch. However, from **Fig. 4c**, we know that 3B05 can still grow and clear acetate even after the growth arrest of 1A01; thus $E_{B2} > E_{A1}$. But from **Supp.**

Fig. 4b, we note that if acetate concentrations are around the concentration where 1A01 starts dying (E_{A2}), 3B05 stops growing and is ineffective in clearing acetate; thus $E_{B2} < E_{A2}$. In our model, we simply take E_{B2} to be at the mid-point of E_{A1} and E_{A2}, i.e., 3.5 mM. We note that all of the hard transitions of our model can be softened without any qualitative differences in the results, and we attempted to use hard transitions as much as possible for parameter parsimony and model simplicity.

Dynamics of the internal variable σ_A. The last factor determining transitions in physiological states for 1A01 is the internal state variable, σ_A. There is not much experimental data to base on here since the cause of the lag in growth arrest is not fully worked out. We do know that the cause is acetate stress, which occurs for $E > E_{A1}$, and the duration of the lag is proportional to the exposure to acetate. As a first approximation, we take the increase of σ_A to be proportional to the acetate concentration integrated over time, with a proportionality constant σ_A^{str}, such that higher stress over longer times lead to a higher value of σ_A. Further, once the acetate concentration falls, i.e., $E \leq E_{A1}$, 1A01 slowly recovers if GlcNAc is present. We take the recovery dynamics of σ_A to have a Michaelis-Menten dependence on GlcNAc concentration with a proportionality constant σ_A^{lag}.

The value of σ_A^{str} was chosen to ensure that σ_A reached a high enough value during acetate stress, and subsequently the values of σ_A^{lag} and σ_A^{c} were chosen such that the total lag time was between 6 and 10 hours as found in **Fig. 4a**. In the simulation, we used rounded parameter values that correspond to a lag time of 8 hours. The results of the simulations did not depend on the exact values of the parameters chosen as long as the resulting lag time was in the range of 4 to 12 hours.

$$\frac{d\sigma_A}{dt} = \begin{cases} \sigma_A^{str}\, E, & E_{A1} < E \text{ and } \sigma_A < 1 \\ -\sigma_A^{lag} f\left(G/K_{A,G}\right), & E \leq E_{A1} \text{ and } \sigma_A > 0 \\ 0, & \sigma_A \geq 1 \text{ or } \sigma_A \leq 0. \end{cases} \tag{3.16}$$

Our complete model is described by Eqs. (3.1)-(3.5) and Eq. (3.16), with the rate functions defined by the entries in **Supplementary Table 5** and **6**, and with the parameters used for the simulations specified in **Supplementary Table 7**. As discussed above, most of the parameters are either fixed by our experimental results (as is the case for most of the maximal growth rates, the yield parameters, the threshold acetate values, etc.), or do not affect the results significantly (as is the case for the Monod constants, σ_A^{str}, etc.) However, as we described following Eqs. (3.8a-c), the parameters associated with the important metabolite-assisted growth of 3B05 at intermediate pH, r_B^+, E_{B2}, and ΔE, were only loosely constrained by experiments. In our simulation, we chose these parameters to take on rounded values that lie within the experimental constraints and recover the observed behaviors, such that 3B05 is able to recover 1A01 in the stable cycle, but is unable to do so in the transient first cycle of a 1:1 initial ratio coculture. The ability of our model to recover the salient features of both the stable cycle and the transient dynamics with a rough approximation for a complex process (metabolite-induced growth under stress) indicates that the emergent qualitative and quantitative features of the model are not very sensitive to model details. However, further experimental studies will be required to elucidate how internal metabolite-assisted growth takes place under acetate stress, to establish the form of the growth function and its associated parameters (Eqs. (3.8a-c)) which are currently proposed phenomenologically.

Supplementary Note 4. Dynamics of the stable cycle and its approach from initial conditions.

We now describe the resulting dynamics of our model of environment-dependent cross-feeding, as defined by Eqs. (3.1)-(3.5) and (3.16) in **Supplementary Note 3** (with the rate functions given by **Supplementary Table 5** and **6**, and the parameters given by **Supplementary Table 7**), from numerical simulations. Outputs of the model are shown in **Supp. Fig. 9** for two initial strain density ratios 3B05:1A01=1:1 and 3:1. We describe here the dynamics of the stable cycle and the transient dynamics for each initial ratio leading to the stable cycle. We also illustrate the dynamics of the internal variable σ_A used in the model to capture the effect of metabolite depletion on growth recovery by 1A01 (**Supplementary Note 3**).

The final cycle attained for initial densities of both 1:1 and 3:1 is the stable cycle depicted in **Fig. 15a**. This cycle illustrates the dynamic nature of the coexistence of 1A01 and 3B05. Starting from ~8 hours into the cycle, the system is in the "Growth Phase" (shaded green region) where both 1A01 and 3B05 grow at their normal rates (solid and dashed black lines, respectively, top plot), as there is abundant GlcNAc (dashed blue line, bottom plot); the pH is in the normal range (acetate, dotted red line bottom plot) below the threshold values indicated by the red horizontal lines above which growth stops; and the internal state of 1A01 (σ_A, solid magenta line in top plot) is below the threshold value (σ_A^c, dotted horizontal magenta line) above which growth stops. In this phase, 1A01 cells are in state A_I^- and 3B05 cells are in state B_I (as defined in **Fig. 6a**).

As 1A01 grows rapidly in the "Growth Phase", acetate quickly accumulates in the coculture. This leads to the growth arrest of 1A01 (entering state A_{II}) when the acetate concentration exceeds the threshold E_{A1}. In this state, 1A01 continues to consume GlcNAc, turning it into metabolites which are then excreted. Additionally, the internal variable σ_A increases rapidly, reflecting the loss of other metabolites due to acetate accumulation (**Fig. 5d-f**). This phase is labelled as the "Stress Phase" and shaded pink. Here 3B05 is in state B_{II} and continues to grow, albeit slowly, assisted by the acetate-induced metabolites excreted from 1A01. The growth of 3B05 slowly removes acetate from the medium.

When the acetate concentration falls below the threshold E_{B1}, 3B05 is back in state B_I and can resume normal growth. It grows even faster than in the Growth Phase due to the presence of acetate-induced metabolites from 1A01 (**Supp. Fig. 6**). This phase is thus called the "Surge Phase" and is shaded as yellow in **Fig. 15a**. 1A01 is no longer inhibited by acetate either in this phase. But it cannot grow for two reasons: First, GlcNAc is depleted, and second, its internal variable σ_A is large. It is in state A_I^+. Since acetate and acetate-induced metabolites are no longer produced by 1A01 in this phase (as GlcNAc is depleted), while 3B05 continues to consume them, eventually, 3B05 clears the acetate and stops growing. The co-culture stagnates at this point and enters the "Wait Phase" (not shaded).

The coculture remains in the "Wait Phase" until dilution into fresh medium in the new cycle. With the infusion of fresh GlcNAc, 1A01 starts its slow recovery by reducing the internal variable σ_A while consuming GlcNAc, mimicking the replenishment of depleted metabolites inside the cell. 1A01 would remain in state A_I^+ as long as $\sigma_A > \sigma_A^c$ ("Lag Phase", purple shade). During this time, 1A01 continues to convert GlcNAc into acetate and acetate-induced metabolites, which support

the growth of 3B05 (in state B_I). When 1A01 is finally able to grow again (when σ_A drops below the threshold σ_A^c ~8 hours later), the system enters the Growth Phase again and the cycle repeats.

While the experimental observations of stable cycle are explained by the dynamical model, we find that the model can even describe the approach to the stable cycle. We first explore the case with 3:1 initial ratio of 3B05 to 1A01 (**Fig. 15b**): In the first cycle, both 1A01 and 3B05 grow at their normal rates from time 0, when there is abundant GlcNAc and normal pH ("Growth Phase", green shaded region). This leads to acetate accumulation and stoppage of growth of 1A01 ("Stress Phase", pink shaded region) as described for the stable cycle in Panel a. The internal variable σ_A also increases rapidly, indicating a loss of metabolites. The high initial ratio of 3B05 to 1A01 ensures that there is enough 3B05 to clear the acetate excreted by 1A01, until the point when GlcNAc is almost depleted, after which the coculture stagnates (Wait Phase, unshaded). Because the first cycle does not have a Lag Phase (as in the stable cycle shown in Panel a), the coculture has a prolonged Wait Phase. Starting from the second cycle, the Lag Phase occupies the first ~8 hours of the cycle, putting the coculture in the stable cycle.

We now explore the approach to the stable cycle of the 1:1 initial ratio of 1A01 and 3B05, which is more complex but still entirely described by the model. In the first cycle (**Fig. 15c**), 1A01 and 3B05 grow when there is abundant GlcNAc, and normal pH as described in panels a and b ("Growth Phase', green shaded region). However, the lower initial density of 3B05 makes it unable to clear acetate rapidly, so that 1A01 enters growth arrest with a lot of GlcNAc still remaining ("Stress Phase", pink shaded region). In the Stress Phase, 1A01 continues to consume GlcNAc and excrete acetate and internal metabolites. Because 3B05 grows slowly and hence consumes acetate slowly in the Stress Phase, acetate continues to accumulate, such that the system is pushed into very high acetate concentrations where 3B05 is in state B_{III} and cannot grow, and 1A01 is in state A_{III} and dies ("Death Phase", cyan shade). In this phase, the density of 1A01 drops rapidly. The cycle also ends with a very high value of the internal variable σ_A indicating a severe loss of metabolites.

At the start of the second cycle for a 1:1 initial ratio of 1A01 and 3B05 (**Fig. 15d**), 1A01 stops dying as acetate is cleared out by dilution. However, due to the large value of σ_A going into the cycle, 1A01 remains in state A_I^+ while its internal state recovers as long as $\sigma_A > \sigma_A^c$, while 3B05 grows on the lingering acetate from the previous cycle and the acetate-induced metabolites excreted by 1A01 as 1A01 is in state A_I^+ ("Lag Phase", purple shade). Once $\sigma_A < \sigma_A^c$, both 1A01 and 3B05 grow exponentially ("Growth Phase", green shade). This Growth Phase lasts much longer than the green phases described in Panels a-c as the starting density of 1A01 is very low, and thus it takes a long time for acetate to accumulate. ~21 hours after the start of the cycle, acetate accumulates enough to stop the growth of 1A01 and 3B05 ("Stress Phase", pink shade). Due to the prolonged period at very low densities, 1A01 is unable to consume all of the GlcNAc by the end of the cycle. Further, because of the short duration in Stress Phase in this cycle, 3B05 is unable to grow and clear the acetate accumulated during the Growth Phase by the end of the cycle. However, the stress at the end of 2nd cycle does lead to a high value of σ_A and a significant Lag Phase in the subsequent (3rd) cycle, placing the coculture in the stable cycle.

From the detailed analysis of the above two examples, we see that if the initial ratio of 3B05 to 1A01 is high enough, it will enter a stable cycle provided that the duration of the cycle is long

enough to contain the four crucial phases: Lag, Growth, Stress, and Surge. If the initial ratio is lower, then the coculture may be able to recover after 1A01 suffers death for a while.

Supplementary References

1. Cermak, N., Datta, M. Sen & Conwill, A. Rapid, Inexpensive Measurement of Synthetic Bacterial Community Composition by Sanger Sequencing of Amplicon Mixtures. *iScience* **23**, 100915 (2020).
2. Bassler, B. L., Yu, C., Lee, Y. C. & Roseman, S. Chitin utilization by marine bacteria: Degradation and catabolism of chitin oligosaccharides by Vibrio furnissii. *Journal of Biological Chemistry* **266**, 24276–24286 (1991).
3. Keseler, I. M. *et al.* EcoCyc: A comprehensive database of Escherichia coli biology. *Nucleic Acids Res* **39**, 583–590 (2011).
4. Basan, M. *et al.* Overflow metabolism in Escherichia coli results from efficient proteome allocation. *Nature* **528**, 99–104 (2015).
5. Tarmy, E. M. & Kaplan, N. O. Kinetics of Escherichia coli B D-Lactate Dehydrogenase and Evidence for Pyruvate-controlled Change in Conformation. *Journal of Biological Chemistry* **243**, 2587–2596 (1968).
6. Muscarella, M. E. & O'Dwyer, J. P. Species dynamics and interactions via metabolically informed consumer-resource models. *Theor Ecol* **13**, 503–518 (2020).
